# Cold ischemia time exceeding 12 hours is a risk factor for delayed graft function and increased mortality in kidney transplant recipients within the eurotransplant senior program

**DOI:** 10.3389/ti.2026.15840

**Published:** 2026-06-17

**Authors:** Laura Matuschik, Lisa André, Philipp A. Holzner, Dietrich A. Ruess, Yakup Tanriver, Johanna Schneider, Bernd Jänigen

**Affiliations:** 1 Department of General and Visceral Surgery, Section of Transplant Surgery, Faculty of Medicine, Medical Center - University of Freiburg, Freiburg, Germany; 2 German Cancer Consortium (DKTK), Partner Site Freiburg and German Cancer Research Center (DKFZ), Heidelberg, Germany; 3 Department of Medicine IV – Nephrology and Primary Care, Faculty of Medicine, Medical Center - University of Freiburg, Freiburg, Germany

**Keywords:** cold ischemia time, delayed graft function, eurotransplant senior program, kidney transplantation, mortality

## Abstract

With the age of those awaiting kidney transplantation (KTx) increasing, the Eurotransplant Senior Program (ESP) reduces median waiting time by > 1.5 years for patients aged ≥65 compared to standard allocation and doubles life expectancy compared with remaining on dialysis. However, older-donor organs are more vulnerable to prolonged cold ischemia time (CIT). To minimize CIT, both kidneys from one donor are regionally allocated, in our case, to a single transplant center. This study assesses the impact of CIT on delayed graft function (DGF) and long-term outcomes in consecutively transplanted ESP recipients from the same donor. We retrospectively analyzed 208 ESP KTx at Freiburg Transplant Center (1999–2019), focusing on 74 consecutively transplanted kidney pairs (recipients ranked “1” or “2”). DGF incidence was similar for rank 1 and rank 2 recipients. CIT >12 h significantly increased DGF risk versus CIT <8 h (adjusted OR 6.30; 95% CI 1.52–26.06; p = 0.011). Death-censored allograft survival was unaffected, but CIT >12 h tripled mortality risk (adjusted HR 3.19; 95% CI 1.44–7.49; p = 0.005). Consecutive transplantation does not disadvantage the second recipient if CIT remains <12 h. CIT >12 h independently predicts DGF and higher mortality, emphasizing the need to minimize ischemia time.

## Graphical Abstract

**Figure:**
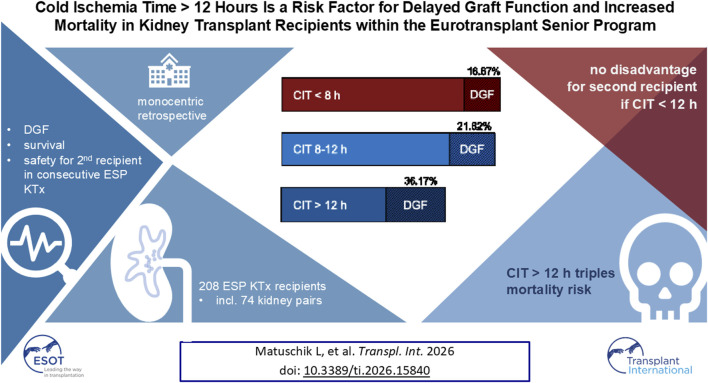

## Introduction

The Eurotransplant Senior Program (ESP) is a 25-year-old initiative providing extended criteria donor (ECD) kidneys to the progressively ageing cohort of listed patients [1, 2]. Its primary objective is to improve transplant opportunities for end-stage renal disease patients aged ≥65 years, who face a fivefold increased mortality risk on the waiting list compared to patients aged <50 years [1, 3]. ESP not only reduces the median waiting time by over 1.5 years compared to standard allocation for patients aged ≥65 years via ETKAS [4, 5] but also doubles life expectancy compared to remaining on dialysis [6–9]. Kidneys from donors aged ≥65 years have been found to perform well despite reduced function, with graft survival comparable to ETKAS-allocated organs for elderly recipients [1, 10–13].

ESP allocates kidneys to primary transplant recipients to mitigate immunologic risk and shortens ischemic organ damage through regional distribution [1]. Cold ischemia time (CIT) is a crucial determinant of graft outcome, especially as older organs are more vulnerable to longer CIT [14, 15]. Ischemic tissue injury triggers an inflammatory response, leading to endothelial activation which, upon reperfusion and infiltration of effector cells, may culminate in acute renal failure, i.e., delayed graft function (DGF) and, ultimately, interstitial fibrosis [16]. Combined with a reduced nephron count in the senescent kidney, the intensified “renal workload” potentially causes hyperfiltration injury [17, 18]. In our specific scenario, the regional allocation frequently results in both kidneys from a single donor being transplanted at our transplant center to two recipients in consecutively performed surgeries.

Studies have shown that reduced CIT improves initial graft function, as evidenced by significantly lower incidences of DGF in the first kidney’s recipient [15, 19]. Additional research confirms CIT as a risk factor for DGF and impaired graft function [4, 11, 20, 21]. A multicenter study by Frei et al. found each hour of CIT raised the risk of graft loss by 3% in ESP-allocated kidneys (RR = 1.03, 95% CI: 1.00–1.05, p = 0.03; mean CIT: 11.9 ± 5.2 h) [4].

Drawing from our clinical experience over the last two decades and contrary to these studies, we did not observe an adverse outcome in the second recipient. This study investigates the impact of CIT on DGF and long-term patient and graft outcomes using data from ESP patients receiving kidneys from the same donor, thus eliminating individual kidney variables.

## Patients and methods

### Patients and study design

This monocentric retrospective study analyses all 208 patients who received a deceased-donor kidney transplant via the ESP at our transplant center over 20 years (27 June 1999 to 27 August 2019), with a mean follow-up of 5.7 years. Approved by the local IRB and registered with the German Clinical Trials Register (protocol 20-1199; registration: DRKS00037372), patients consented to record use for research.

All 208 postmortal transplants were ABO- and Human Leukocyte Antigen (HLA)-compatible, defined as negative complement-dependent cytotoxicity (CDC) crossmatch, with CDC remaining the mandatory pre-transplant compatibility test throughout the study period per local protocol. ESP allocation prioritized ABO blood-group matching and negative crossmatch over HLA matching according to Eurotransplant guidelines. As per center policy, kidneys for immunized patients were only accepted when preformed DSA could be excluded preoperatively.

**FIGURE 1:**
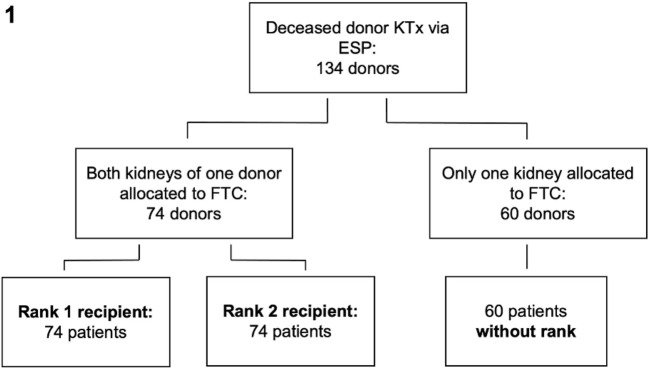
Study profile. 208 patients receiving a deceased-donor kidney transplantation via ESP allocation. KTx: kidney transplantation; ESP: Eurotransplant Senior Program; FTC: Freiburg Transplant Center.

Graft preparation, surgical procedure, and follow-up were as described [22]. All kidneys were preserved by static cold storage (SCS), using Histidine-Tryptophane-Ketoglutarate (HTK) solution (Custodiol®) or University of Wisconsin (UW) solution. Hypothermic machine perfusion (HMP) was not used during the study period (1999-2019), as it was not adopted in the Eurotransplant region until post-2015. Recipients stayed in intermediate care for 5–7 days post-transplant. Figure 1 shows the study profile. In 74 cases, both kidneys from a single donor were allocated to our center and, due to availability of operating theatres and surgeons, transplanted consecutively, with recipients labeled as “rank 1” and “rank 2.” In 60 cases, only one kidney was received, which is included in graft and survival analysis but not in mixed logistic regression.

The patient cohort was stratified by CIT. While a CIT >18 h is reported to impair graft survival, this was rare in our case [23, 24]. With a median CIT of 9.35 h in our cohort, we performed an ROC analysis to determine the optimal CIT cut-offs. We then categorized CIT 1 (0–8 h), CIT 2 (8–12 h), and CIT 3 (equal to or greater than 12 h) according to ROC analysis and clinical practicability.

Clinical and follow-up data were acquired from our nephrological transplant outpatient clinic and the local nephrologists. Delayed graft function was defined as the need for ≥1 dialysis within 7 days postoperatively. PNF was defined as permanent dysfunction requiring dialysis and was excluded from DGF analyses to avoid misclassification. Graft loss was defined as permanently resuming dialysis due to irreversible graft failure. Acute reversible graft failure was excluded.

### Statistical analysis

Results with p-values <0.05 were considered statistically significant and assessed on a two-sided basis. The dataset was tested for normality; results are shown in Supplementary Table S1; Supplementary Figure S1 (Supplementary Material). Categorical data were assessed using Fisher’s exact test; continuous data were assessed using paired t-test, Wilcoxon matched-pairs signed-rank test, Mann-Whitney-U test and Kruskal-Wallis, presented either as mean ± SD or as median and 95% CI.

A post-hoc power analysis was performed to assess the statistical power of the study given the available sample size. For survival outcomes (patient survival and death-censored graft survival), power was calculated using the Schoenfeld formula based on the observed number of events and hazard ratios derived from Cox proportional hazards models. For the comparison of delayed graft function rates between CIT groups, Cohen’s h was used as the effect size measure. All calculations assumed a two-sided significance level of α = 0.05. Statistical power was calculated using the pwr package (version 1.3) in R (version 2024.09.1 + 394).

ROC curve analysis was performed to determine the optimal CIT cut-off for predicting DGF, graft loss, and patient survival. Optimal cut-off was determined by Youden index. Recipients with primary non-function (PNF, n = 15) were excluded (193 recipients remaining).

Delayed graft function (DGF) was the primary endpoint, evaluated through a mixed effects logistic regression with random intercept for donor identity (74 donors) to account for the non-independence of outcomes in kidney pairs from the same donor. The model specification (generalized linear mixed model (GLMM) with binominal distribution and logit link function) was

$$\text{logit}\left(P\left({\text{DGF}}_{\text{ij}}\right)\right)=\beta_{0}+\beta_{1}(\text{CIT}\_\text{group}2{)}_{\text{ij}}+\beta_{2}(\text{CIT}\_\text{group}3{)}_{\text{ij}}+\beta_{3}(\text{DM}{)}_{\text{j}}+\beta_{4}(\text{CHD}{)}_{\text{j}}+\beta_{5}(\text{HT}{)}_{\text{j}}+\beta_{6}(\text{Age}{)}_{\text{j}}+\beta_{7}(\text{BMI}{)}_{\text{j}}+u_{j}$$

where “i” denotes the recipient (1 or 2) and “j” denotes the donor (1–74).

“CIT_group2” and “CIT_group3” are indicator variables for CIT 8–12 h and >12 h, respectively (reference: CIT 0–8 h). We included the following donor-specific covariates: diabetes mellitus (DM), coronary heart disease (CHD), hypertension (HT), age (Age), and body mass index (BMI). The model was fitted using maximum likelihood estimation with the Laplace approximation in R (lme4 package, version 1.1–35).

Secondary endpoints included graft and patient survival, analyzed via the Kaplan-Meier method and log-rank test, considering the paired cohort (148 patients) for graft survival (functional integrity of individual graft) and all 208 patients for patient survival (limited sample size, donor-specific characteristics not as important), focusing on a five-year follow-up. PNF was included as immediate failure (time = 0). Cox proportional hazards regression assessed CIT impact on secondary endpoints. The regression model included confounder adjustments based on clinical relevance and biological plausibility. Goodness-of-fit was tested with Log-likelihood ratio, Wald, and Score tests. Multicollinearity was checked using variance inflation factors (Supplementary Table S5).

To assess whether the long inclusion period (1999–2019) influenced study findings, a sensitivity analysis was performed. Patients were divided into three transplant eras: Era 1 (1999–2006, ESP introduction), Era 2 (2007–2013, tacrolimus, refined surgical techniques), and Era 3 (2014–2019, modern protocols). Eras were chosen according to major milestones and improvements in transplant protocols. First, the transplant era was included as a covariate in all multivariable Cox regression models. Second, a formal interaction test between the CIT group and transplant era was performed using a likelihood ratio test comparing models with and without the interaction term.

Missing data were minimal (<4% for any variable). Specifically, primary immunosuppression (n = 2, 0.96%), creatinine at discharge (n = 7, 3.37%), warm ischemia time (n = 1, 0.48%), time on dialysis before KTx (n = 1, 0.48%), and time on wait list (n = 1, 0.48%) were missing due to incomplete historical records (see STROBE Statement in Supplementary Material). Complete case analysis was used for multivariable models. All primary outcomes (DGF, graft loss, and death) had complete data with no loss to follow-up for the five-year observation period, as all patients were followed at our center and at the local nephrologists.

All analyses and visualizations were conducted using GraphPad Prism 11 and R version 2024.09.1 + 394.

## Results

### Baseline characteristics

Among the 208 patients who received an ESP allograft at our transplant center, in 74 cases both kidneys from a single donor were transplanted consecutively in two patients, designated as rank 1 and rank 2 recipients. Comparing these two groups, no differences were observed with respect to sex, body mass index (BMI), ASA classification, pre-existing medical conditions, time on dialysis prior to KTx, time on the wait list, or the number of HLA mismatches (Table 1A; for a more detailed comparison of groups, refer to Supplementary Table S2). As a result of consecutively performed transplantations, the cold ischemic period was significantly prolonged in rank 2 recipients (10.7 (9.8, 11.5) vs. 6.3 (5.6, 7.1) h, p < 0.0001, Table 1A). Similar characteristics were noted when comparing patients according to CIT groups (CIT groups): no significant differences in demographics, comorbidities, or HLA mismatches, except CIT, were found (Table 1B, for post-hoc pairwise comparisons for baseline characteristics of CIT groups see Table 1C).

**TABLE 1:** Baseline characteristics.

(A)	Rank 1 Recipient	Rank 2 Recipient	p-value
​	n = 74	n = 74	​
Recipients' characteristics			
Female sex, n (%)	23 (31.1)	19 (25.7)	0.585
Age at transplantation (years)	66 (66, 68)	67 (66, 68)	0.747
BMI, recipient (kg/m^2^)	25.72 (±3.47)	26.17 (±3.36)	0.389
ASA category	3 (3, 3)	3 (3, 3)	0.524
Pre-existing medical conditions, n (%)	​	​	—
Diabetes mellitus	21 (28.4)	13 (17.6)	0.171
Hypertension	38 (51.4)	37 (50.0)	>0.999
Coronary heart disease	31 (41.9)	33 (44.6)	0.868
Asthma	0 (0)	2 (2.7)	0.497
COPD	3 (4.1)	3 (4.1)	>0.999
Time on dialysis before KTx (days)	2010 (±786.4)	1885 (±832.3)	0.227
Time on wait list (days)	1189 (983, 1523)	1129 (866, 1415)	0.720
Total number of mismatches	5 (4, 5)	4 (4, 5)	0.400
A mismatches	1 (1, 2)	1 (1, 2)	0.410
B mismatches	2 (2, 2)	1.5 (1, 2)	0.201
DR mismatches	2 (1, 2)	2 (1, 2)	0.281
PRA level (≥5%), n (%)	11 (14.9)	18 (24.3)	0.147
Surgical data			
Duration of surgery (h)	2.6 (2.4, 2.8)	2.6 (2.4, 2.9)	0.320
Ischemia time, total (h)	6.8 (6.0, 7.6)	11.1 (10.1, 12.2)	<0.0001
Cold ischemia time (h)	6.3 (5.6, 7.1)	10.7 (9.8, 11.5)	<0.0001
Warm ischemia time (min)	29 (26, 30)	30 (28, 33)	0.368

(B)	CIT Group 1	CIT Group 2	CIT Group 3	p-value
​	n = 76	n = 78	n = 54	​
Recipients' characteristics				
Female sex, n (%)	25 (32.9)	26 (33.3)	8 (14.8)	0.033
Age at transplantation (years)	67 (66, 68)	67 (66, 68)	68 (66, 71)	0.834
BMI, recipient (kg/m2)	25.8 (24.6, 26.9)	25.7 (24.7, 26.9)	25.8 (25.0, 26.5)	0.918
ASA category	3 (3, 3)	3 (3, 3)	3 (3, 3)	0.407
Pre-existing medical conditions, n (%)	​	​	​	—
Diabetes mellitus	22 (28.9)	13 (16.7)	16 (29.6)	0.118
Hypertension	31 (40.8)	40 (51.3)	22 (40.7)	0.342
Coronary heart disease	29 (38.2)	31 (39.7)	26 (48.1)	0.490
Asthma	1 (1.3)	2 (2.6)	0 (0)	0.784
COPD	5 (6.6)	3 (3.8)	5 (9.3)	0.389
Time on dialysis before KTx (days)	1875 (1710, 2040)	1770 (1470, 2010)	1665 (1380, 1980)	0.169
Time on wait list (days)	1329 (1045, 1625)	1029 (726, 1403)	915 (631, 1203)	0.139
Total number of mismatches	4 (4, 5)	4 (4, 5)	4 (4, 5)	0.218
A mismatches	1 (1, 2)	1 (1, 1)	1 (1, 2)	0.128
B mismatches	2 (2, 2)	1 (1, 2)	2 (1, 2)	0.193
DR mismatches	2 (1, 2)	1 (1, 2)	1 (1, 2)	0.537
PRA level (≥5%), n (%)	11 (14.5)	16 (20.5)	9 (16.7)	0.623
Surgical data				
Duration of surgery (h)	2.7 (2.5, 3.0)	2.6 (2.2, 2.7)	2.6 (2.2, 2.8)	0.114
Ischemia time, total (h)	6.2 (5.5, 6.9)	10.3 (9.9, 11.0)	14.9 (13.9, 15.7)	<0.0001
Cold ischemia time (h)	5.8 (5.1, 6.3)	9.8 (9.4, 10.4)	14.4 (13.2, 15.0)	<0.0001
Warm ischemia time (min)	29 (27, 30)	30 (27, 31)	30 (28, 33)	0.882

(C) Comparison	p-value	Significant (p < 0.017)?
Female sex		
CIT 1 vs. CIT 2	p > 0.9999	No
CIT 1 vs. CIT 3	p = 0.024	No
CIT 2 vs. CIT 3	p = 0.025	No
Ischemia time, total		
CIT 1 vs. CIT 2	p < 0.0001	Yes
CIT 1 vs. CIT 3	p < 0.0001	Yes
CIT 2 vs. CIT 3	p < 0.0001	Yes
Cold ischemia time		
CIT 1 vs. CIT 2	p < 0.0001	Yes
CIT 1 vs. CIT 3	p < 0.0001	Yes
CIT 2 vs. CIT 3	p < 0.0001	Yes

A: Baseline characteristics of rank 1 and rank 2 recipients. p-values from paired t-test and Wilcoxon test (Mann-Whitney U test when missing data). For normality testing results see Supplementary Table S1. B: Baseline characteristics by CIT group. CIT group 1: 0–8 h; CIT group 2: 8–12 h; CIT group 3: >12 h p-values from Fisher’s exact test and Kruskal-Wallis test. Data presented as n (%) for categorical variables, mean (±SD) for normally distributed continuous variables, or median (95% CI of median) for non-normally distributed continuous variables. C: Post-hoc Pairwise Comparisons (Bonferroni Correction) for baseline characteristics of CIT groups. Pairwise comparisons performed using Fisher’s exact test and Mann-Whitney U test with Bonferroni correction. Adjusted significance threshold: p < 0.017 (0.05/3 comparisons). BMI = Body Mass Index; ASA = American Society of Anesthesiologists; COPD = Chronic Obstructive Pulmonary Disease; KTx = Kidney Transplantation.

### Delayed graft function

Among 193 transplants with initial function, DGF occurred in 45 cases (23.3%). Cases with primary non-function (PNF, n = 15) were excluded. Among 74 kidney pairs, DGF was 20% for rank 1% and 30% for rank 2 recipients and 18.9% in single kidney allocations (kidneys with PNF excluded: rank 1: 4 kidneys; rank 2: 4 kidneys; single kidneys: 7 kidneys). No significant difference was observed concerning extended dialysis (>7 days post-transplant) (Table 2).

**TABLE 2:** A: ROC curve analysis to determine the optimal cold ischemia time (CIT) cut-off for predicting DGF, graft loss, and patient survival. AUC = Area Under the Curve. Optimal cut-off determined by Youden index. Recipients with primary non-function (PNF, n = 15) were excluded (193 recipients remaining). B: Composition of patient groups based CIT and KTx rank position. Data presented as n (%). Percentages for CIT subgroups calculated within each CIT group. p-values from Fisher’s exact test; †p-value from chi-square test for CIT group distribution*.* In 74 instances, both kidneys of one donor were transplanted consecutively in two recipients at our transplant center (rank 1 and rank 2). ‘Single kidney’: in 60 instances, only one kidney was allocated to our transplant center (right column). Kidneys with primary non-function (PNF) were excluded. CIT: cold ischemia time. C. Post-hoc Pairwise Comparisons (Bonferroni Correction) for CIT group composition. Pairwise comparisons performed using Chi-square test with Bonferroni correction. Adjusted significance threshold: p < 0.017 (0.05/3 comparisons).

(A) Outcome	AUC (95% CI)	p-value	Optimal CIT Cut-off	Sensitivity/Specificity
Delayed graft function	0.592 (95% CI 0.497–0.687)	p = 0.031	8.53 h	71.1%/48%
Graft loss	0.597 (95% CI 0.443–0.750)	p = 0.115	11.3 h	50%/73.7%
Patient death	0.63 (95% CI 0.504–0.756)	p = 0.014	11.6 h	57.1%/79.4%

(B)	Rank 1 Recipient	Rank 2 Recipient	Single Kidney	p-value
​	n = 74	n = 74	n = 60	​
COMPOSITION OF PATIENT GROUPS				
Primary non-function, n (%)	4 (5.4)	4 (5.4)	7 (11.7)	p = 0.311
Patients per CIT group, n (%)	*n = 70*	*n = 70*	*n = 53*	—
CIT 1: 0–8 h	50 (71.4)	8 (11.4)	14 (26.4)	**p < 0.0001†**
CIT 2: 8–12 h	14 (20.0)	38 (54.3)	22 (41.5)	—
CIT 3: >12 h	6 (8.6)	24 (34.3)	17 (32.1)	—
Delayed graft function, n (%)	14 (20.0)	21 (30.0)	10 (18.9)	p = 0.286
CIT 1 (n, % of CIT group)	8 (15.1)	1 (12.5)	3 (20.0)	—
CIT 2	4 (26.7)	9 (23.1)	3 (12.5)	—
CIT 3	2 (33.3)	11 (40.7)	4 (19.0)	—
Dialysis >7 days postoperative, n (%)	13 (17.6)	15 (20.3)	15 (25.0)	p = 0.661

(C) Comparison	p-value	Significant (p < 0.017)?
Rank 1 vs. rank 2	p < 0.0001	Yes
Rank 1 vs. single kidney	p < 0.0001	Yes
Rank 2 vs. single kidney	p = 0.0889	No

The bold value means the statistically significant.

**FIGURE 2:**
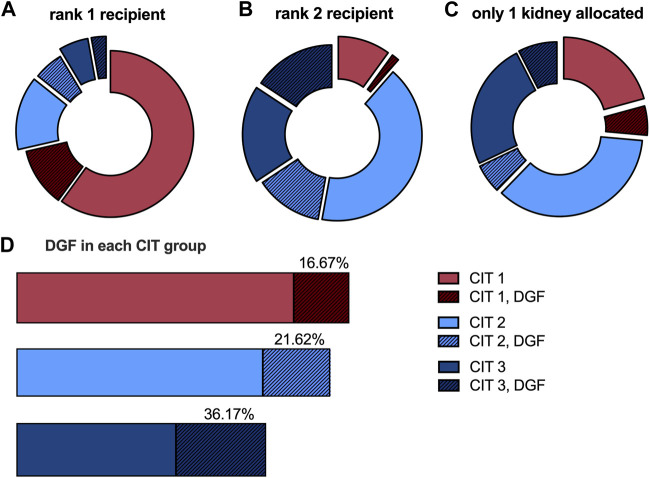
Distribution of CIT groups and proportions of DGF for 193 patients receiving a graft allocated through the ESP. Shown are the compositions of each recipient group **(A–C)** and the respective proportions of DGF for each CIT group **(D)**. CIT 1: 0–8 h; CIT 2: 8–12 h; CIT 3: >12 h. Kidneys with primary non-function were excluded (n = 15). **(A)** Rank 1 recipients (n = 70). **(B)** Rank 2 recipients (n = 70). **(C)** Only one kidney allocated to our transplant center (n = 53). **(D)** Proportions of DGF for each CIT group (whole cohort, n = 193, PNF excluded). CIT: cold ischemia time, DGF: delayed graft function; ESP: Eurotransplant Senior Program.

CIT was the only significant difference between rank 1 and rank 2 (Table 1A). Subsequently, the entire cohort was stratified into CIT groups (Table 2; Figures 2A–C), with ROC curve analysis aiding in identifying the optimal CIT cut-off for predicting DGF (Table 2A, for ROC curves and their comparison see Supplementary Material; Supplementary Figure S2). CIT demonstrated poor discriminative ability for DGF with an AUC of 0.592 (95% CI 0.497–0.687, p = 0.031). The optimal cut-off was determined using the Youden index, yielding a CIT threshold of 8.53 h, with a sensitivity of 71.1% and specificity of 48%. To take the most important secondary endpoints into account as well, we added ROC curve analyses for graft and patient survival. We found that CIT demonstrated poor discriminative ability for graft loss with an AUC of 0.597 (95% CI 0.443–0.750, p = 0.115). The optimal cut-off was determined using the Youden index, yielding a CIT threshold of 11.3 h, with a sensitivity of 50% and specificity of 73.7%. Concerning patient survival, CIT demonstrated acceptable discriminative ability with an AUC of 0.63 (95% CI 0.504–0.756, p = 0.014). The optimal cut-off was determined using the Youden index, yielding a CIT threshold of 11.6 h, with a sensitivity of 57.1% and specificity of 79.4%. With the median CIT being 9.35 h in our cohort, we set the groups as CIT 1 (0–8 h), CIT 2 (8–12 h), and CIT 3 (≥12 h), taking both clinical practicability and ROC analysis results into account.

Although KTx were performed consecutively, nearly two-thirds (63.5%) of rank 2 recipients received the allograft within 12 h (Table 2B). DGF proportions were similar in CIT groups 1 and 2 regardless of rank (rank 1 (CIT <12 h): 18.8%, rank 2 (CIT <12 h): 21.7%; Table 2B). When stratifying the cohort according to CIT, by every 4-h increment, DGF rates rose steadily, from 16.7% (CIT <8 h), to 21.6% (CIT 8–12 h), up to 36.2% (CIT >12 h; Figure 2D).

A mixed logistic regression analysis examined the relationship between CIT and DGF. To mitigate potential confounding factors pertaining to allograft characteristics, analysis was restricted to the 74 kidney pairs and further adjustment for donors’ pre-existing medical conditions was made (Table 3A). Kidneys with PNF were excluded from analysis. CIT group 3 had a 6.3-times higher risk of DGF than CIT group 1 (adjusted OR 6.30; 95% CI 1.52–26.06; p = 0.011, Table 3B). Additionally, the risk of DGF increased with donor age (OR: 1.23; CI: 1.05–1.43; p = 0.01, Table 3B). To underscore the need for minimizing CIT, the analysis was replicated with CIT measured in minutes: DGF still correlated with increasing CIT (OR: 1.0002; CI: 1.0–1.01; p = 0.012; Supplementary Table S3A), although not as pronounced as with categorical groups. Rank of transplantation did not affect DGF (Supplementary Table S3B).

**TABLE 3:** Relative risk of DGF post-KTx via ESP allocation.

(A)	All Donors
​	n = 74
Donor characteristics	
Female sex, n (%)	34 (45.9)
Age at transplantation (years)	69 (68, 70)
BMI (kg/m^2^)	25.85 (24.7, 26.1)
Pre-existing medical conditions, n (%)	
Diabetes mellitus	11 (14.9)
Hypertension	41 (55.4)
Coronary heart disease	11 (14.9)
Cause of death, n (%)	​
Traumatic head injury	17 (23.0)
Cerebral ischemia	11 (14.9)
Intracranial bleeding	46 (62.2)
Resuscitation, n (%)	6 (8.1)

(B) Predictor Variable	Odds Ratio	95% CI	p-value
Outcome: Delayed graft function (DGF)			
CIT group 2 (ref: CIT group 1)	2.19	0.73–6.58	0.164
CIT group 3 (ref: CIT group 1)	**6.30**	1.52–26.06	**0.011**
Donors' pre-existing conditions			
Diabetes mellitus	1.07	0.22–5.10	0.933
Coronary heart disease	1.00	0.17–5.75	0.998
Hypertension	0.63	0.22–1.77	0.378
Age (per year)	**1.23**	1.05–1.43	**0.010**
BMI (per kg/m^2^)	0.97	0.84–1.12	0.704

(A) Donor characteristics with both kidneys allocated to our transplant center. Data presented as n (%) for categorical variables or median (95% CI of median) for continuous variables. BMI = Body Mass Index. (B) Relative risk of DGF of matched allografts by CIT groups (CIT group 1 served as reference; group 1: 0–8 h; group 2: 8–12 h; group 3: >12 h); kidneys with primary non-function (4 kidneys for each rank) were excluded, adjusted for potentially confounding pre-existing medical conditions of the donors. BMI: body mass index, CHD: coronary heart disease, CI: confidence interval, CIT: cold ischemia time, DGF: delayed graft function, OR: Odds ratio. The bold value means the statistically significant.

### Graft function and patient survival

After identifying CIT as a risk factor for DGF, rather than the chronological sequence of graft transplantation, we analyzed its impact on graft function and patient survival. Creatinine levels were similar at discharge and last follow-up (Supplementary Table S4). Graft loss incidence was similar across CIT groups but occurred earlier in CIT group 3 (57 (1, 639) days vs. 674 (129, 1664) days, p = 0.042, Supplementary Table S4).

**FIGURE 3:**
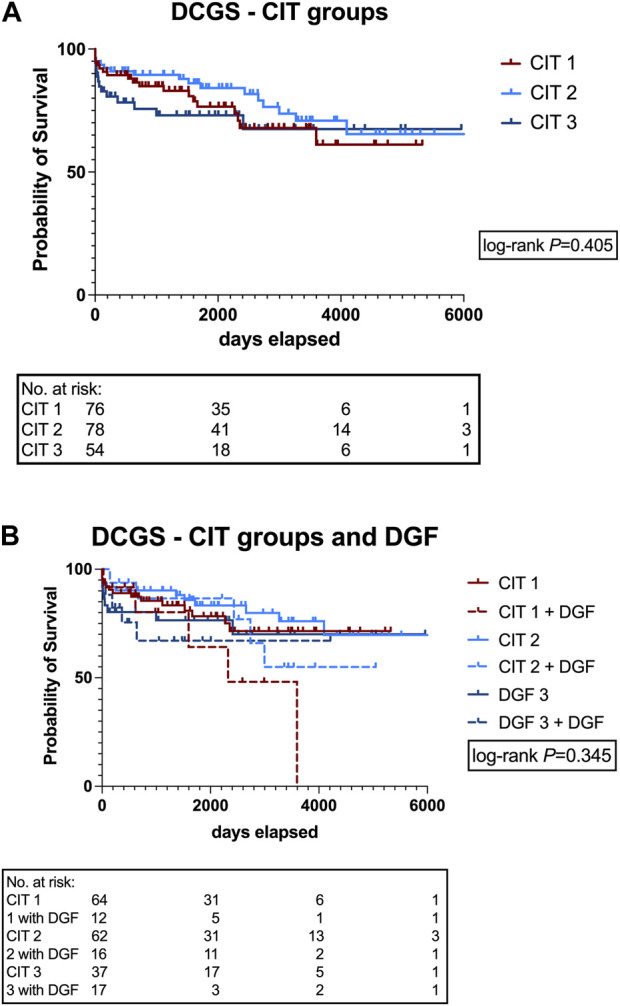
Death-censored graft survival post-ESP-KTx by CIT group and DGF. **(A)** Death-censored graft survival. **(B)** Death-censored graft survival with CIT groups divided by DGF status. CIT 1: 0–8 h; CIT 2: 8–12 h; CIT 3: >12 h. Data for 208 patients. Kaplan-Meier graph with log-rank test used for graft loss. CIT: cold ischemic time; DGF: delayed graft function.

In matched patients without DGF, graft survival rates were comparable among CIT groups (Figure 3A). For those with DGF, CIT group 3 exhibited a numerically (though not statistically significant, p = 0.345) lower five-year graft survival rate (67.1% vs. CIT 1: 78.4%/CIT 2: 83.3%), which may reflect insufficient power (Figure 3B).

Cox multivariable regression analysis identified DGF as the only independent risk factor for graft loss (adjusted HR: 3.71, CI: 1.14–12.53, p = 0.029, Table 4), meaning CIT, transplantation rank (Supplementary Figure S3) and recipients’ comorbidities were not risk factors.

**TABLE 4:** Death-censored graft survival post-ESP-KTx. Relative hazard of death-censored graft loss by risk factors, shown for 148 matched patients.

(4) Predictor Variable	Hazard Ratio	95% CI	p-value
Outcome: Graft loss			
Rank (rank 2 vs. rank 1)	0.99	0.28–3.87	0.991
CIT group 2 (ref: CIT group 1)	0.92	0.20–4.17	0.918
CIT group 3 (ref: CIT group 1)	0.95	0.18–4.84	0.954
Delayed graft function (DGF)	**3.71**	1.14–12.53	**0.029**
ASA 3 (ref: ASA 1–2)	0.56	0.16–2.59	0.399
ASA 4 (ref: ASA 1–2)	2.53	0.11–24.37	0.458
Age at transplantation >70 years (R)	0.28	0.04–1.20	0.134
Male sex (R)	1.01	0.29–4.00	0.992
Posttransplant infection	3.02	0.96–11.63	0.075

Model diagnostics are provided in Supplementary Material (Supplementary Table S5). ASA: American Association of Anesthesiologists; CI: confidence interval; CIT: cold ischemic time; DGF: delayed graft function; KTx: kidney transplantation; R: recipient. The bold value means the statistically significant.

**FIGURE 4:**
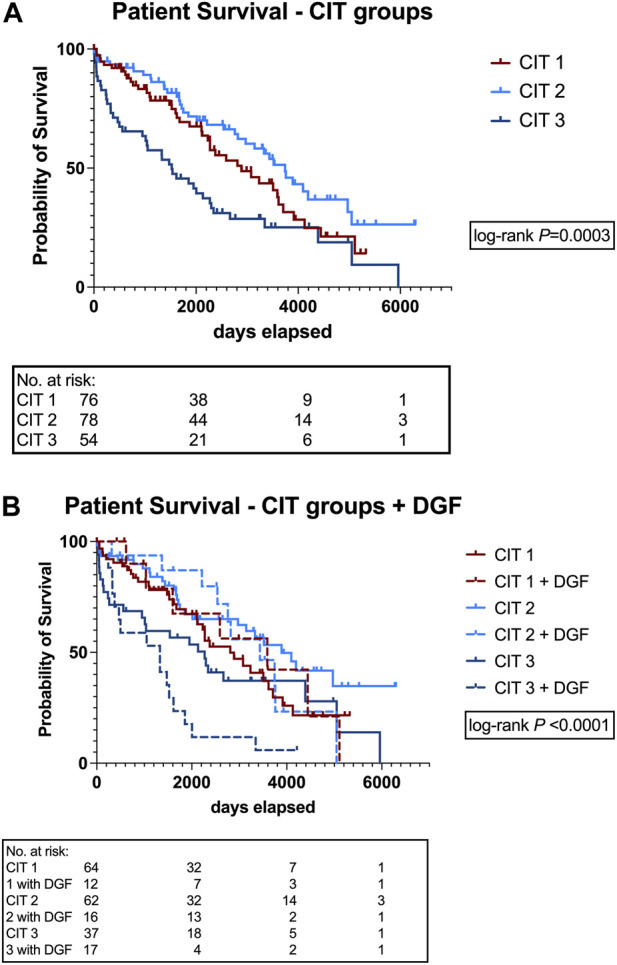
Patient survival post-ESP-KTx by CIT group and DGF. **(A)** Overall survival. **(B)** Patient survival by CIT groups divided by DGF status. CIT 1: 0–8 h; CIT 2: 8–12 h; CIT 3: >12 h. Data for 208 patients. Kaplan-Meier graph with log-rank test used for survival. CIT: cold ischemic time; DGF: delayed graft function.

Patient survival was assessed in the whole cohort. Kaplan-Meier analysis indicated higher mortality in CIT group 3 (log-rank P = 0.0003, Figure 4A), whereas survival rates between rank 1 and rank 2 were comparable (Supplementary Figure S3). DGF worsened outcomes in CIT group 3 (log-rank P < 0.0001, Figure 4B), with a 23.5% five-year survival rate that was markedly lower than the other groups (CIT 1: 69.5%, with DGF: 67.5%; CIT 2: 69.4%, with DGF: 87.1%; CIT 3: 56.7%).

To understand why survival within CIT group 3, even without DGF, was significantly worse, we compared postoperative complications. Significantly more cardiac complications were seen in CIT group 3 (9.1% vs. 22.2%, p = 0.017, Table 5). Nonetheless, using a Cox proportional hazards regression model, the sole independent risk factor for impaired survival remained a CIT >12 h, increasing mortality by 2.98 times (adjusted HR 2.98, 95% CI: 1.34–6.97, p = 0.009, Table 6).

**TABLE 5:** Postoperative complications by CIT groups.

​	CIT Groups 1 and 2	CIT Group 3	p-value
​	N = 154	N = 54	​
Non-infectious complications			
Allograft rejection, n (%)	52 (33.8)	19 (35.2)	0.850
Lymphocele, n (%)	19 (12.3)	5 (9.3)	0.542
Needing operative revision	15 (78.95)	4 (80.0)	0.960
Postoperative bleeding/hematoma, n (%)	27 (17.5)	10 (18.5)	0.871
Needing operative revision	19 (70.4)	7 (70.0)	0.983
Postoperative vessel occlusion/TRAST, n (%)	10 (6.5)	2 (3.7)	0.449
Requiring operative/interventional revision	6 (60.0)	1 (50.0)	0.793
Postoperative urological complication, n (%)	41 (29.9)	17 (31.5)	0.527
Requiring operative/interventional revision	21 (51.2)	10 (58.8)	0.597
Postoperative cardiac complication, n (%)	14 (9.1)	12 (22.2)	**0.012**
Post-KTx diabetes/hyperglycemic imbalance, n (%)	14 (9.1)	6 (11.1)	0.665
Infectious complications			
Postoperative infection, n (%)	73 (47.4)	29 (53.7)	0.426
Urinary tract infection, n (%)	64 (87.7)	21 (75.0)	0.119
*Urosepsis*	7 (10.9)	1 (4.8)	0.400
Pneumonia, n (%)	11 (7.1)	6 (11.1)	0.360
*Pulmonary sepsis*	2 (18.2)	2 (33.3)	0.482
Sepsis with unknown focus, n (%)	2 (22.2)	4 (57.1)	0.152
Postoperative wound healing disorder, n (%)	24 (15.6)	14 (25.9)	0.091
CMV	​	​	—
CMV status of donor positive	85 (55.2)	38 (70.4)	0.051
CMV status of recipient negative	64 (41.6)	16 (29.6)	0.121
Risk constellation (D+/R−)	35 (22.7)	12 (22.2)	0.076
CMV positivity	15 (9.7)	10 (18.5)	0.087

CIT group 1 and 2: cold ischemia time ≤12 h; CIT group 3: cold ischemia time >12 h. Data shown for 208 patients with median values (95% CI of median) unless indicated otherwise. CMV: cytomegalovirus; KTx: kidney transplantation; TRAST: transplant renal artery stenosis/thrombosis. The bold value means the statistically significant.

**TABLE 6:** Patient survival post-ESP-KTx.

Predictor Variable	Hazard Ratio	95% CI	p-value
Outcome: Patient survival			
CIT group 2 (ref: CIT group 1)	0.615	0.21–1.68	0.354
CIT group 3 (ref: CIT group 1)	**2.980**	1.34–6.97	**0.009**
Delayed graft function (DGF)	1.100	0.45–2.47	0.824
ASA 3 (ref: ASA 1–2)	2.943	1.01–12.55	0.082
ASA 4 (ref: ASA 1–2)	0.984	0.05–7.99	0.989
Age at transplantation >70 years (R)	0.773	0.33–1.70	0.535
Female sex (R)	1.154	0.52–2.85	0.739
Postoperative infection	1.159	0.56–2.41	0.688
Allograft rejection	2.063	0.76–4.99	0.126
Cardiac complication	0.873	0.39–1.89	0.735
Urological complication	0.894	0.38–1.93	0.784
Postoperative bleeding	0.595	0.19–1.51	0.316

Relative hazard of death post-KTx by risk factors, with model diagnostics provided in Supplementary Material (Supplementary Table S5). CIT group 2: 8–12 h; CIT group 3: >12 h. Data for 208 patients. ASA: American Association of Anesthesiologists; CI: confidence interval; CIT: cold ischemic time; DGF: delayed graft function; R: recipient characteristic. The bold value means the statistically significant.

To address the potential confounding effect of the long inclusion period, a sensitivity analysis was performed. After adjustment for transplant era (Era 1: 1999-2006, Era 2: 2007-2013, Era 3: 2014-2019), the association between CIT group 3 and patient survival remained significant (HR = 4.81, 95% CI: 1.21–19.99, p = 0.025, Supplementary Table S6). No significant interaction between CIT group and transplant era was observed (Likelihood ratio test (Era × CIT interaction): patient survival: p = 0.583; death-censored graft survival: p = 0.254, Supplementary Table S6), indicating that the prognostic effect of prolonged CIT was consistent across all three transplant eras.

**FIGURE 5:**
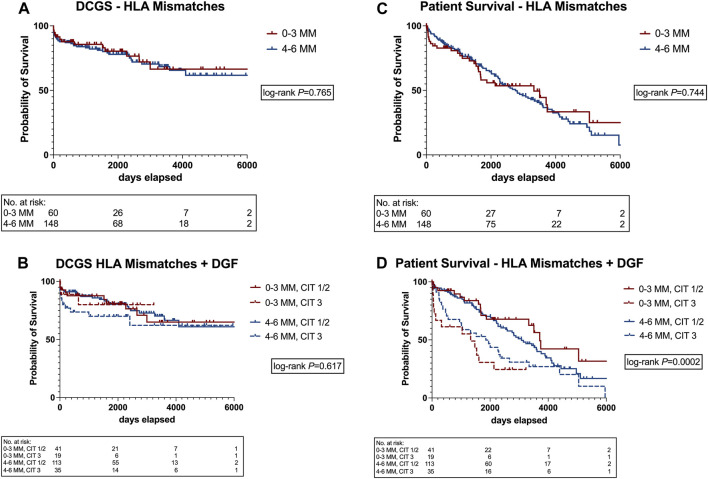
Allograft and patient survival post-ESP-KTx by number of mismatches and CIT. **(A)** Death-censored graft survival by mismatches. **(B)** Death-censored graft survival by mismatches and CIT. **(C)** Patient survival by mismatches. **(D)** Patient survival by mismatches and CIT groups. CIT1/2: cold ischemia time ≤12 h. CIT 3: cold ischemia time >12 h. KTx: kidney transplantation. MM: mismatches.

HLA-DR matching for ESP patients has been advocated for, with de Fijter et al. positing it enhances five-year graft and patient survival [25]. Accordingly, we assessed outcomes of our Freiburg ESP cohort considering HLA-mismatches. Among 28 patients with zero HLA-DR mismatches, 21% experienced DGF, matching the whole cohort’s proportion (21.6%). Kaplan-Meier analysis showed no survival differences between patients with 0–3 and 4-6 mismatches (Figures 5A,C). However, differentiating mismatch groups by CIT, CIT group 3 patients, irrespective of mismatches, had lower survival compared to CIT groups 1 and 2 (log-rank P = 0.0002, Figure 5D). This trend was also present for death-censored graft survival, though the analysis did not achieve statistical significance (log-rank P = 0.617, Figure 5B). A comparable analysis of the 74 kidney pairs yielded consistent findings (Supplementary Figure S4).

To supplement this retrospective analysis, a Cox proportional hazards regression model identified risk factors for mortality, adjusting for confounders like ASA category and DGF. Regardless of mismatch count, CIT >12 h tripled mortality risk (Table 7A: CIT 3: adjusted HR 2.89, 95% CI: 1.33–6.65, p = 0.009; Table 7B: CIT 3: adjusted HR 3.07, 95% CI: 1.43–6.97, p = 0.005).

**TABLE 7:** Relative hazard of death post-ESP-KTx by risk factors, including mismatches.

(A) Predictor Variable	Hazard Ratio	95% CI	p-value
Outcome: Patient survival			
Delayed graft function (DGF)	0.947	0.41–1.99	0.890
DR mismatch 1 (ref: MM DR 0)	0.602	0.23–1.69	0.313
DR mismatch 2 (ref: MM DR 0)	0.749	0.32–1.96	0.526
CIT group 2 (ref: CIT group 1)	0.592	0.20–1.60	0.310
CIT group 3 (ref: CIT group 1)	**2.893**	1.33–6.65	**0.009**
ASA category 3 (ref: ASA 1–2)	2.515	0.89–10.52	0.129
ASA category 4 (ref: ASA 1–2)	0.761	0.04–5.98	0.813

(B) predictor variable	Hazard ratio	95% CI	p-value
Outcome: Patient survival			
Delayed graft function (DGF)	0.948	0.41–1.99	0.893
Total mismatches (MM)	0.953	0.74–1.25	0.719
CIT group 2 (ref: CIT group 1)	0.588	0.20–1.59	0.306
CIT group 3 (ref: CIT group 1)	**3.067**	1.43–6.97	**0.005**
ASA category 3 (ref: ASA 1–2)	2.556	0.90–10.74	0.125
ASA category 4 (ref: ASA 1–2)	0.786	0.04–6.15	0.835

Data are shown for all 208 patients. (A) Relative hazard of death after KTx taking DGF, HLA-DR mismatches (MM DR), CIT, and ASA category into account. (B) Relative hazard of death after KTx taking DGF, the total number of HLA-mismatches, CIT, and ASA category into account. Model diagnostics are provided in Supplementary Material (Supplementary Table S5). ASA: American Association of Anesthesiologists; CI: confidence interval; CIT: cold ischemic time; DGF: delayed graft function; KTx: kidney transplantation; MM: mismatches; R: recipient. The bold value means the statistically significant.

Post-hoc power analysis revealed that the study was underpowered for several comparisons, with power ranging from 11.9% to 68.3% across outcomes, primarily reflecting the limited number of events inherent to this low-volume transplant program (Supplementary Table S7).

## Discussion

This clinical study investigates the influence of CIT on the outcomes of expanded criteria donor kidneys using a paired kidney ‘case-control’ design in the largest cohort to date, aiming to clarify the safety of consecutive KTx within the ESP, particularly for the second recipient.

Despite rank 2 recipients encountering a substantially longer CIT compared to their rank 1 counterparts, the rank position itself did not adversely influence initial graft function. Previous research also found transplantation sequence does not impact post-transplant outcomes. Subgroup analyses within two retrospective single-center studies on 10 and 20 ESP kidney pairs with a similar CIT (rank 1: 7 h/7.6 h; rank 2: 12 h/13.4 h, compared to 6.3 h vs. 10.7 h in our cohort) observed no difference in rejection rates, DGF, functional data, or long-term graft and patient survival [26, 27]. In contrast, Krüger et al. examined 5 ESP kidney pairs and reported that rank 1 recipients (CIT: 5.5 ± 2.0 h) experienced no DGF, while 3 out of 5 rank 2 recipients (CIT: 11.7 ± 3.1 h) needed dialysis post-transplant, suggesting ‘ultra-short’ CIT benefits early graft function in marginal donors [15]. Our data show DGF rates rise with CIT time; for every 4-h increment of CIT, DGF rates grow by approximately 10% within our paired kidney cohort, ranging from roughly 15% for kidneys with a CIT less than 8 h, to 25% in the 8–12 h CIT range, and up to 35% for kidneys with a CIT exceeding 12 h. A CIT exceeding 12 h multiplies the risk of DGF six-fold compared to under 8 h. A cohort analysis within the U.S. Renal Data System database echoed this, with the risk of DGF elevating by 23% for every 6-h increase in CIT [28]. These results suggest that focus should be oriented toward reducing CIT rather than the sequence of KTx in a paired transplant setting.

DGF was found to affect overall graft function and long-term transplant outcomes. It was the sole independent risk factor for graft loss in our cohort, evidenced by a hazard ratio of 3.71. When coupled with a CIT >12 h, grafts with DGF exhibited a reduced five-year graft survival rate of 67.1% compared to those with shorter ischemic times, aligning with several studies highlighting DGF as a key predictor of graft loss over a five-year period [20, 28]. Heldal et al. confirm that DGF predicts death-censored graft loss across all age groups but notably among recipients aged >70 years (adjusted HR: 3.96 (95% CI: 1.38–11.37)) [29].

In efforts to quantify the risk of graft failure associated with ischemia time, two dynamics have been proposed: a linear increase by 3% per hour of CIT [4]; and a progressive risk rise surpassing a CIT >18 h, with a relative risk (RR) of 1.09 for periods between 19–24 h and an RR of 1.36 for periods exceeding 36 h [24]. Similarly, a study on ECD kidney outcomes using Collaborative Transplant Study data found that a CIT of less than 18 h did not significantly impair five-year survival, even in ECD kidneys aged 70 years and older [23]. Nonetheless, the outcomes reported for ECDs ≥70 years were derived from recipients with an average age of 64 years, with approximately 40% of these recipients being under 65 years of age, thus rendering a comparison with our notably older patient cohort (mean age: 68.4 years) challenging. While prolonged CIT correlates with impaired graft function, it is plausible that additional factors, especially in older cohorts, may also impact graft outcomes. We found that advancing donor age serves as a pertinent factor in the development of DGF, whereas other studies cite recipient age as a determinant influencing graft outcome [30].

KTx doubles the life expectancy of elderly end-stage renal disease patients compared to remaining on dialysis, although an improvement in survival was only observed after the first post-transplantation year had passed [6, 7]. Our survival analysis produced consistent results for rank 1 and rank 2 recipients, aligning with several other studies [26, 31]. Notably, we found an increased mortality rate in the group with a CIT exceeding 12 h, particularly pronounced in DGF patients, showing a 23.5% survival rate at 5 years post-transplant. While considering cohort comorbidities, such as a higher rate of cardiac complications in CIT group 3, prolonged CIT (>12 h) emerged as the most significant predictor of mortality. Unlike the findings from Heldal et al. linking rejection episodes and their treatment to higher mortality, our study found no increased rejection rate with prolonged CIT [29].

In reference to ESP allocation regulations, which prioritize a shorter CIT over HLA matching, concerns exist on whether the incorporation of HLA-DR matching could improve outcomes. Our study found HLA mismatches had no impact on graft or patient survival within the first 2 years post-transplant. Within our cohort, fewer mismatches did not reduce the impact of long ischemia time, with CIT being the only independent predictor of increased mortality. Literature is inconsistent about HLA matching’s effect on ESP outcomes. Our results are corroborated by several studies, indicating that the ESP patients’ suboptimal histocompatibility did not increase rejection or immunological graft loss, even with a mean CIT of 23.6 ± 8.6 h and 31.1% DGF incidence [20, 32]. Furthermore, data from the U.S. Renal Data System showed higher five-year graft survival for kidneys with HLA mismatches but without DGF than for zero-mismatched kidneys with DGF (63% vs. 51%) [28]. These findings imply that ischemic injury and perioperative conditions may exert a more substantial influence on graft function than HLA matching.

Conversely, a number of European studies underscores the potential benefits of HLA-DR matching in reducing T-cell mediated rejections and enhancing long-term outcomes [33]. Doxiadis et al. reported that complete HLA-DR compatibility decreased acute rejection within the first 6 months post-transplant and substantiated their findings through an analysis of Eurotransplant data, which demonstrated superior graft survival for HLA-DR compatible transplants [34]. The Eurotransplant Senior DR-compatible Program (ESDP) indicated that zero HLA-DR mismatches significantly lower five-year mortality risk (HR 0.71, 95% CI: 0.53–0.95) and decrease graft failure and return to dialysis risks at one and 5 years [25]. While the ESDP group had a longer CIT (12 (9-15) h vs. 11 (8-13) h), median waiting time for ESDP patients was notably reduced compared to the ESP group (2.6 (1.8–4.0) years vs. 4.2 (2.8–5.5) years) [25]. Considering that shorter waiting time on dialysis may contribute to ESDP’s success, its focus on a zero DR-mismatch raises concerns about equitable organ allocation, since not all candidates on the wait list might achieve such a match [35]. Immunized and repeat KTx patients were excluded from the study protocol, leaving it unclear if they would benefit from DR-matching. Our results align with literature emphasizing the importance of CIT and DGF in transplant outcomes, highlighting the need for improved organ preservation and perioperative management, regardless of HLA matching.

Focusing on molecular changes during cold ischemia, ATP depletion, coupled with the lack of glycogen and oxygen, leads to tubular epithelial cell injury, contributing to vascular leakage and interstitial oedema [36, 37]. Machine perfusion (MP) has been studied as an alternative to cold storage to reduce tissue injury, especially in ECD kidneys. Hypothermic machine perfusion (HMP) was not routinely used during the study period at our center for ESP kidneys. The decision to use static cold storage (SCS) rather than HMP reflected temporal factors (HMP was not widely adopted in the Eurotransplant region until after 2015) and logistical considerations (consecutive transplantation of both kidneys from the same donor within a short timeframe precluded pump allocation to different preservation modalities). Comparative studies of ESP or ECD kidney pairs show MP advantages in preventing DGF and primary non-function, as well as reducing the risk of graft failure [38–40]. However, Kox et al. suggest that the damaging effect of CIT during MP is similar to cold storage, increasing DGF risk by 8% per hour, with ischemic oedema affecting capillary flow equally in both MP and cold storage [37]. While HMP may mitigate inflammatory responses by the elimination of proinflammatory cytokines, shear stress may increase vascular resistance, leading to vascular injury over prolonged MP durations [37, 41, 42].

In January 2026, the German Organ Procurement Organization (DSO) implemented HMP for ECD kidney transplants on a nationwide scale, which may modify CIT thresholds. These developments present an opportunity to revisit the ongoing discourse on HLA matching for KTx recipients within the ESP and potentially reassess its benefits coupled with MP.

Although this analysis currently represents the largest patient collective comparing outcomes of consecutively transplanted ESP kidneys, it has several important limitations. First, the monocentric design limits external validity and generalizability. Second, our cohort consists predominantly of white European patients, limiting applicability to other ethnic groups. Third, retrospective data collection over 20 years (1999–2019) introduces inherent biases related to temporal changes in immunosuppression, surgical techniques, and perioperative care. However, our era-specific sensitivity analyses demonstrate that the CIT effect remained consistent across three distinct time periods, supporting robustness of findings despite practice evolution. Fourth, the retrospective single-center design and the inherently limited patient volume of the ESP program resulted in relatively small group sizes, particularly for CIT group 3. Post-hoc power analysis confirmed that several comparisons were below the conventional 80% power threshold, most notably for graft survival outcomes. This increases the risk of Type II error, and non-significant findings should therefore be interpreted with caution. Nonetheless, this cohort represents one of the largest systematic analyses of CIT effects within the ESP, and the significant associations identified are considered robust given the conservative statistical approach applied. Fifth, all donors were DBD; findings may not generalize to DCD kidneys with inherently higher DGF rates and potentially different CIT sensitivity. Finally, generalizability to non-Eurotransplant regions (UNOS, UK) may be limited due to different allocation algorithms, geographical distances, and preservation standards.

In conclusion, this study demonstrates that, within the analyzed cohort, CIT emerged as the primary determinant of DGF and patient survival, over factors such as transplantation sequence or HLA mismatches. The reduction of ischemia time continues to be imperative for enhancing outcomes, particularly in the context of ECD kidneys. Our findings provide concrete guidance for ESP kidney allocation and surgical planning. When both kidneys from a single ESP donor are allocated to one center, surgical coordination should prioritize completing both transplantations within 12 h of procurement. This may require parallel operating rooms, availability of two surgical teams, or preferential early-day scheduling. When CIT >12 h is unavoidable, patient counseling should include discussion of increased DGF and mortality risk. Importantly, our data demonstrate that rank 2 recipients are not disadvantaged if CIT remains <12 h, which may inform recipient selection and consent discussions. The implementation of HMP in Germany provides an opportunity to re-evaluate whether the 12-h threshold can be safely extended under improved preservation conditions.

## Data Availability

The original contributions presented in the study are included in the article/Supplementary Material, further inquiries can be directed to the corresponding author.
