# Supplementary material for: Cold ischemia time exceeding 12 hours is a risk factor for delayed graft function and increased mortality in kidney transplant recipients within the eurotransplant senior program
Source: Transpl Int. 2026 Jun 17;39:15840. doi: 10.3389/ti.2026.15840 (PMC13318802; doi:10.3389/ti.2026.15840)
Supplement: Supplementary file 1 [file DataSheet1.pdf]

# Supplemental Digital Content

**Table S1. Normality testing results.** Normality assessed using the Shapiro-Wilk test for each group separately (Rank 1 recipient and Rank 2 recipient). p-values $< 0.05$ indicate rejection of normality. Variables with both groups passing normality ($p > 0.05$) were analyzed using parametric tests; all others were analyzed using non-parametric tests. KTx = Kidney Transplantation; BMI = Body Mass Index; ASA = American Society of Anesthesiologists.

| Variable | Shapiro-Wilk p-value | | Assumed Distribution | Statistical Test Used |
|---|---|---|---|---|
| | *Rank 1* | *Rank 2* | | |
| Duration of hospitalization (days) | p<0.0001 | p<0.0001 | Non-normal | *Wilcoxon matched-pairs signed rank test* |
| Creatinine at discharge (mg/dl) | p<0.0001 | p<0.0001 | Non-normal | *Mann-Whitney test (due to missing values)* |
| Creatinine at last follow-up (mg/dl) | p<0.0001 | p<0.0001 | Non-normal | *Mann-Whitney test (due to missing values)* |
| Age at transplantation, recipient (years) | p<0.0001 | p<0.0001 | Non-normal | *Wilcoxon matched-pairs signed rank test* |
| BMI, recipient (kg/m$^2$) | p=0.2276 | p=0.1077 | Normal | *Paired t-test* |
| ASA category | p<0.0001 | p<0.0001 | Non-normal | *Wilcoxon matched-pairs signed rank test* |
| Time on dialysis before KTx (days) | p=0.5720 | p=0.9717 | Normal | *Paired t-test* |
| Time on wait list (days) | p=0.0262 | p=0.0006 | Non-normal | *Wilcoxon matched-pairs signed rank test* |
| Total number of mismatches | p<0.0001 | p<0.0001 | Non-normal | *Wilcoxon matched-pairs signed rank test* |
| A mismatches | p<0.0001 | p<0.0001 | Non-normal | *Wilcoxon matched-pairs signed rank test* |
| B mismatches | p<0.0001 | p<0.0001 | Non-normal | *Wilcoxon matched-pairs signed rank test* |
| DR mismatches | p<0.0001 | p<0.0001 | Non-normal | *Wilcoxon matched-pairs signed rank test* |
| Duration of surgery (h) | p=0.0411 | p=0.0106 | Non-normal | *Wilcoxon matched-pairs signed rank test* |
| Ischemia time, total (h) | p<0.0001 | p=0.1007 | Non-normal | *Wilcoxon matched-pairs signed rank test* |
| Cold ischemia time (h) | p<0.0001 | p=0.1117 | Non-normal | *Wilcoxon matched-pairs signed rank test* |
| Warm ischemia time (min) | p=0.0124 | p=0.0558 | Non-normal | *Wilcoxon matched-pairs signed rank test* |

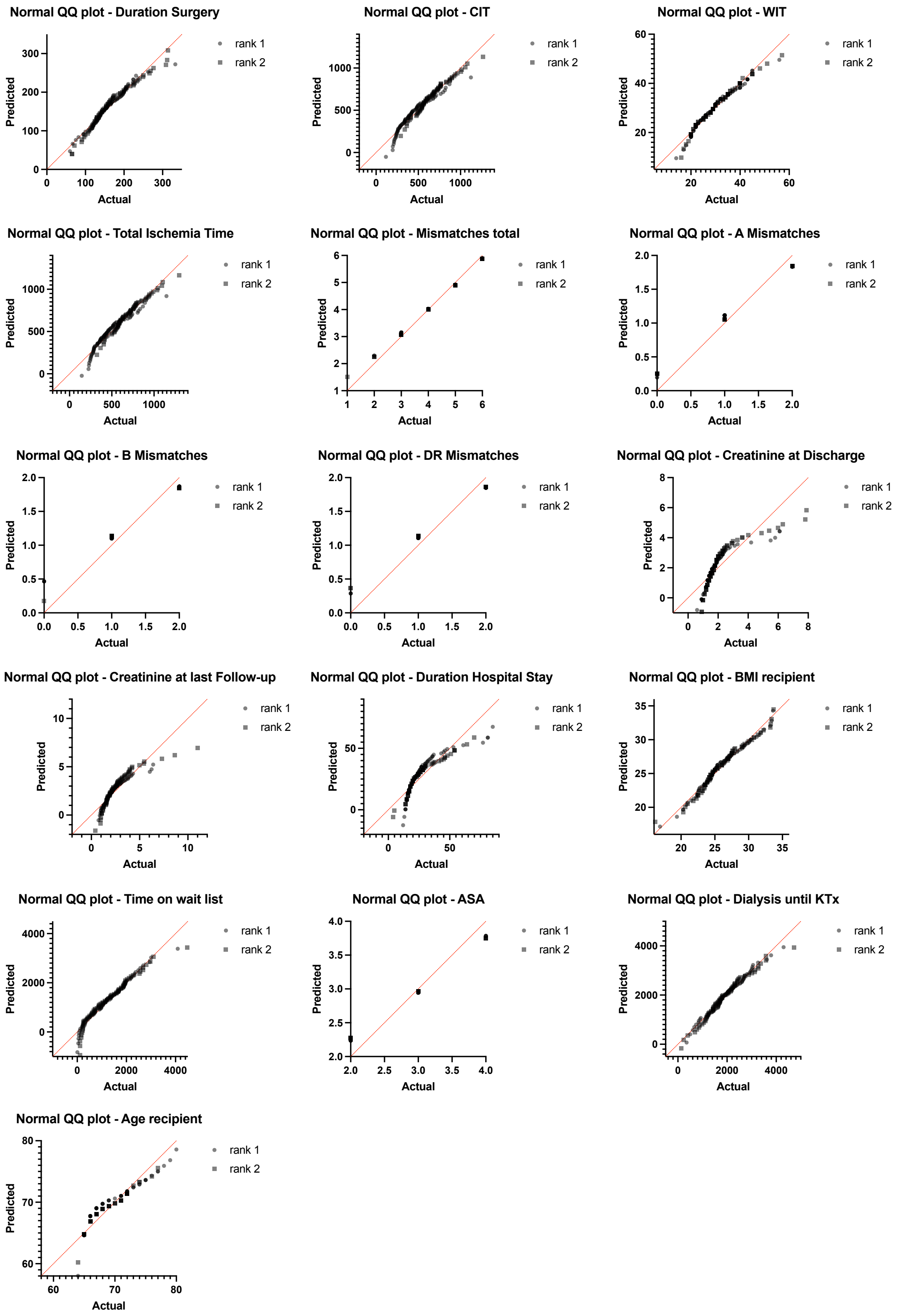


**Figure S1. Normality testing results.** Normality assessed using Q-Q plots for each group separately (Rank 1 recipient and Rank 2 recipient). KTx = Kidney Transplantation; BMI = Body Mass Index; ASA = American Society of Anesthesiologists; WIT = warm ischemia time, CIT = cold ischemia time.

**Table S2. Baseline characteristics and outcomes of rank 1 and rank 2 recipients.** Data presented as n (%) for categorical variables or median (95% CI of median) for continuous variables. *Two rank 1 and four rank 2 patients underwent two simultaneous surgeries. +One rank 1 and two rank 2 patients suffered from more than one infection. Percentages calculated within patients with postoperative infection. KTx = Kidney Transplantation; CAPD = Continuous Ambulatory Peritoneal Dialysis; PRA = Panel Reactive Antibody; MMF = Mycophenolate Mofetil; ICU = Intensive Care Unit; TRAST = Transplant Renal Artery Stenosis/Thrombosis; DJ = Double-J ureteral stent; CMV = Cytomegalovirus; D = donor; R = recipient.

| | **Rank 1 Recipient** *n=74* | **Rank 2 Recipient** *n=74* |
|---|---|---|
| **RECIPIENTS' CHARACTERISTICS** | | |
| **Blood group, recipient, n (%)** | — | — |
| A | 32 (43.2) | 34 (45.9) |
| AB | 0 (0) | 1 (1.4) |
| B | 6 (8.1) | 8 (10.8) |
| 0 | 36 (48.6) | 31 (41.9) |
| **Primary disease, n (%)** | — | — |
| Glomerulonephritis | 26 (35.1) | 26 (35.1) |
| Polycystic kidney disease | 13 (17.6) | 22 (29.7) |
| Hypertension | 4 (5.4) | 4 (5.4) |
| Diabetic nephropathy | 9 (12.2) | 8 (10.8) |
| Other | 22 (29.7) | 14 (18.9) |
| Number of KTx > 1, n (%) | 13 (17.6) | 23 (31.1) |
| Dialysis before KTx, n (%) | 74 (100) | 74 (100) |
| Time from first dialysis until KTx (days) | 1931 (1736, 2207) | 1780 (1537, 2077) |
| PRA level (≥ 5%), n (%) | 11 (14.9) | 18 (24.3) |
| **IMMUNOLOGICAL DATA** | | |
| **Initial immunosuppression regime, n (%)** | — | — |
| Ciclosporin + MMF + prednisone | 15 (20.3) | 17 (23.0) |
| Tacrolimus + MMF + prednisone | 50 (67.6) | 51 (68.9) |
| Prednisone + MMF | 5 (6.8) | 4 (5.4) |
| Tacrolimus + MMF | 2 (2.7) | 1 (1.4) |
| Ciclosporin + Tacrolimus + MMF + prednisone | 1 (1.4) | 0 (0) |
| Tacrolimus + prednisone | 1 (1.4) | 1 (1.4) |

| | Rank 1 Recipient<br>*n=74* | Rank 2 Recipient<br>*n=74* |
|---|---|---|
| **Immunosuppression regime at discharge, n (%)** | — | — |
| Ciclosporin + MMF + prednisone | 16 (21.6) | 14 (18.9) |
| Tacrolimus + MMF + prednisone | 48 (64.9) | 51 (68.9) |
| Prednisone + MMF | 0 (0) | 0 (0) |
| Tacrolimus + MMF | 6 (8.1) | 6 (8.1) |
| Tacrolimus + prednisone | 1 (1.4) | 2 (2.7) |
| Sirolimus + prednisone | 2 (2.7) | 0 (0) |
| No immunosuppression at discharge | 1 (1.4) | 1 (1.4) |
| Change in immunosuppression regime, n (%) | 13 (17.6) | 12 (16.2) |
| Simulect, n (%) | 44 (59.5) | 45 (60.8) |
| Thymoglobulin, n (%) | 12 (14.3) | 8 (9.5) |
| **SURGICAL DATA** | | |
| Patients undergoing simultaneous surgical procedures, n (%) | 22* (29.7) | 31* (41.9) |
| Kidney transplant nephrectomy | 13 (17.6) | 23 (31.1) |
| Explantation of CAPD catheter | 6 (8.1) | 3 (4.1) |
| Other | 5 (6.8) | 9 (12.2) |
| Duration of hospitalization (days) | 22.5 (20, 26) | 22 (20, 26) |
| Requiring therapy on ICU, n (%) | 14 (18.9) | 10 (13.5) |
| Duration of therapy on ICU (days) | 3.5 (2, 9) | 4.5 (2, 13) |
| **NON-INFECTIOUS COMPLICATIONS** | | |
| Allograft rejection, n (%) | 24 (32.4) | 26 (35.1) |
| Postoperative biopsy, n (%) | 21 (28.4) | 28 (37.8) |
| Lymphocele, n (%) | 11 (14.9) | 5 (6.8) |
| Needing operative revision | 8 (72.7) | 5 (100) |
| Postoperative bleeding/hematoma, n (%) | 17 (23.0) | 10 (13.5) |
| Needing operative revision | 13 (76.5) | 8 (80.0) |
| Postoperative vessel occlusion/TRAST, n (%) | 4 (5.4) | 5 (6.8) |
| Requiring operative/interventional revision | 2 (50.0) | 2 (40.0) |
| Postoperative urological complication, n (%) | 23 (31.1) | 22 (29.7) |
| Requiring operative/interventional revision | 12 (52.2) | 13 (59.1) |
| Days until removing of DJ catheter (median) | 15 (14, 18) | 15 (14, 17) |
| Postoperative cardiological complication, n (%) | 9 (12.2) | 8 (10.8) |
| Post-KTx diabetes or hyperglycemic imbalance, n (%) | 6 (8.1) | 7 (9.5) |

| | Rank 1 Recipient<br>*n=74* | Rank 2 Recipient<br>*n=74* |
|---|---|---|
| Allograft nephrectomy, total, n (%) | 5 (6.8) | 5 (6.8) |
| Within first 30 days after KTx | 1 (1.4) | 1 (1.4) |
| Within first year after KTx | 4 (5.4) | 4 (5.4) |
| **INFECTIOUS COMPLICATIONS** | | |
| Postoperative infection, n (%) | 30 (40.5) | 42 (56.8) |
| Urinary tract infection, n (%) | $27^{+}$ (90.0) | $35^{+}$ (83.3) |
| *Urosepsis* | 1 (3.7) | 5 (14.3) |
| Pneumonia, n (%) | $3^{+}$ (10.0) | $6^{+}$ (14.3) |
| *Pulmonary sepsis* | 1 (33.3) | 1 (16.7) |
| Sepsis with unknown focus, n (%) | $1^{+}$ (3.3) | $3^{+}$ (7.1) |
| Postoperative wound healing disorder, n (%) | 14 (18.9) | 13 (17.6) |
| **CMV, n (%)** | — | — |
| CMV status of donor positive | 42 (56.8) | 42 (56.8) |
| CMV status of recipient negative | 28 (37.8) | 25 (33.8) |
| Risk constellation (D+ / R−) | 9 (12.2) | 11 (14.9) |
| CMV positivity | 16 (21.6) | 13 (17.6) |

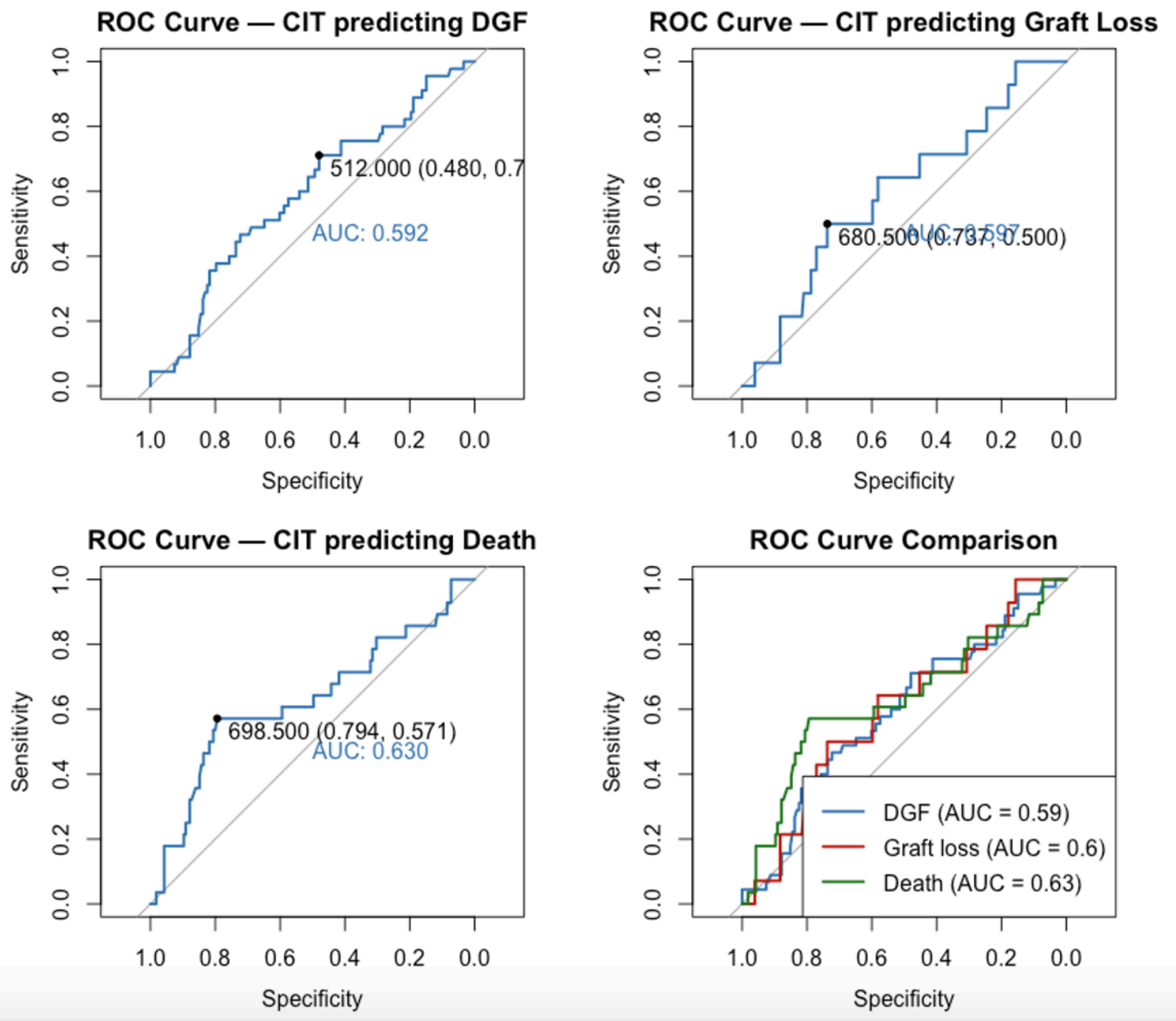


**Figure S2. ROC curve analysis** showing AUC for DGF, graft loss and patient death. Optimal cut-offs for CIT shown in minutes, determined by Youden index.

**Table S3. Relative risk of DGF after KTx with an ESP-allocated allograft.** Mixed logistic regression with kidney pair as random effect. Odds ratios > 1 indicate increased odds of DGF. **(A)** Relative risk based on CIT (minutes) of matched allografts. PNF excluded. **(B)** Relative risk of DGF based on CIT (minutes) of matched allografts including rank of transplantation, PNF excluded. Adjusted for donors' pre-existing medical conditions. BMI: body mass index, CHD: coronary heart disease, CI: confidence interval, CIT: cold ischemia time, DGF: delayed graft function, OR: Odds ratio.

| A Predictors | Odds Ratio | 95% CI | p-value |
|---|---|---|---|
| **OUTCOME: DELAYED GRAFT FUNCTION (DGF)** | | | |
| **CIT (min)** | **1.0002** | 1.00 – 1.01 | **0.012** |
| **Donors' Pre-existing Conditions** | | | |
| Diabetes mellitus | 0.97 | 0.21 – 4.41 | 0.964 |
| Coronary heart disease (CHD) | 0.90 | 0.17 – 4.73 | 0.900 |
| Hypertension | 0.58 | 0.22 – 1.58 | 0.289 |
| **Age (per year)** | **1.20** | 1.04 – 1.39 | **0.012** |
| BMI (per $kg/m^2$) | 0.98 | 0.86 – 1.13 | 0.803 |

| B Predictors | Odds Ratio | 95% CI | p-value |
|---|---|---|---|
| **OUTCOME: DELAYED GRAFT FUNCTION (DGF)** | | | |
| CIT (min) | 1.00 | 1.00 – 1.01 | **0.083** |
| Rank (rank 2 vs rank 1) | 1.13 | 0.40 – 3.21 | 0.812 |
| **Donors' Pre-existing Conditions** | | | |
| Diabetes mellitus | 0.97 | 0.21 – 4.40 | 0.966 |
| Coronary heart disease (CHD) | 0.88 | 0.17 – 4.68 | 0.880 |
| Hypertension | 0.59 | 0.22 – 1.58 | 0.291 |
| **Age (per year)** | **1.20** | 1.04 – 1.39 | **0.012** |
| BMI (per $kg/m^2$) | 0.98 | 0.86 – 1.13 | 0.802 |

**Table S4. Long-term outcome based on CIT groups.** Median values are provided (95% CI of median) unless indicated otherwise. Percentages for cause of graft loss and cause of death calculated within patients who experienced graft loss or death, respectively. CIT group 1 + 2: cold ischemia time ≤ 12 h; CIT group 3: cold ischemia time > 12 h. KTx: kidney transplantation.

| | CIT Groups 1 and 2 | CIT Group 3 |
|---|---|---|
| | *N=154* | *N=54* |
| **LONG-TERM OUTCOME** | | |
| Creatinine at discharge (mg/dl) | 1.7 (1.6, 1.9) | 1.8 (1.6, 2.0) |
| Creatinine at last follow-up (mg/dl) | 2.1 (1.8, 2.3) | 2.2 (2.0, 3.1) |
| **COMPLICATIONS** | | |
| **Graft loss, n (%)** | 31 (20.1) | 12 (22.2) |
| Days from KTx to graft loss (median) | 674 (129, 1664) | 57 (1, 639) |
| Graft loss in first 2 years after KTx, n (%) | 17 (11.0) | 12 (22.2) |
| **Cause of graft loss, n (%)** | — | — |
| *Infectious* | 6 (19.4) | 4 (33.3) |
| *Thrombotic/insufficient perfusion* | 3 (9.7) | 2 (16.7) |
| *Chronic rejection* | 4 (12.9) | 1 (8.3) |
| *Unknown/other* | 18 (58.1) | 5 (41.7) |
| **Death, n (%)** | 76 (49.4) | 40 (74.1) |
| Death with functioning allograft, n (%) | 30 (39.5) | 14 (35.0) |
| Death within first 2 years after KTx, n (%) | 16 (21.1) | 18 (45.0) |
| *With functioning allograft, n (%)* | 6 (37.5) | 7 (38.9) |
| **Cause of death, n (%)** | — | — |
| *Infection* | 34 (44.2) | 17 (42.5) |
| *Cancer* | 9 (11.8) | 5 (12.5) |
| *Cardiovascular* | 9 (11.8) | 8 (20.0) |
| *Unknown/other* | 24 (31.6) | 10 (25.0) |

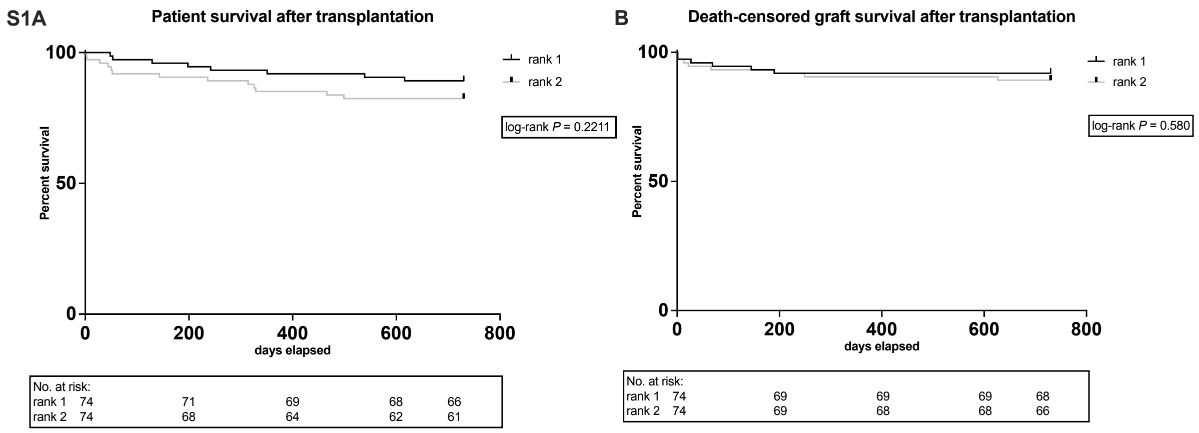


**Figure S3. Survival rates for patients and grafts survival within the first two years post-KTx by transplantation rank. (A)** Patient survival. **(B)** Death-censored graft survival. Data for 148 patients (74 kidney pairs). Kaplan-Meier graph with log-rank test used for graft loss and death. KTx: kidney transplantation.

**Table S5. Cox proportional hazard regression models with model diagnostics. (A)** graft loss by clinically relevant risk factors, AIC: 132.3 (empty model AIC: 139.2). **(B)** patient survival by clinically relevant risk factors, AIC: 350.8 (empty model AIC: 354.4). **(C)** patient survival by risk factors including HLA-DR mismatches, AIC: 354.5 (empty model AIC: 348.0). **(D)** patient survival by risk factors including total mismatches, AIC: 354.5 (empty model AIC: 346.9). All models' hypothesis tests (Log-likelihood ratio test, Wald test, Score test) rejected the null hypothesis. AIC: Akaike's information criterion; VIF: variance inflation factors.

| A Predictor Variable | $\beta$ | HR | 95% CI | p-value | VIF |
|---|---|---|---|---|---|
| **OUTCOME: GRAFT LOSS** | | | | | |
| Rank (rank 2 vs rank 1) | -0.007 | 0.99 | 0.28 – 3.87 | 0.991 | 3.40 |
| CIT group 2 (ref: CIT group 1) | -0.078 | 0.92 | 0.20 – 4.17 | 0.918 | 2.58 |
| CIT group 3 (ref: CIT group 1) | -0.048 | 0.95 | 0.18 – 4.84 | 0.954 | 2.34 |
| **Delayed graft function (DGF)** | 1.312 | **3.71** | 1.14 – 12.53 | **0.029** | 1.41 |
| ASA category 3 (ref: ASA 1–2) | -0.580 | 0.56 | 0.16 – 2.59 | 0.399 | 3.16 |
| ASA category 4 (ref: ASA 1–2) | 0.926 | 2.53 | 0.11 – 24.37 | 0.458 | 1.18 |
| Age >70 years (R) | -1.256 | 0.28 | 0.04 – 1.20 | 0.134 | 1.52 |
| Male sex (R) | 0.007 | 1.01 | 0.29 – 4.00 | 0.992 | 2.94 |
| Posttransplant infection | 1.106 | 3.02 | 0.96 – 11.63 | 0.075 | 1.93 |

| B Predictor Variable | $\beta$ | HR | 95% CI | p-value | VIF |
|---|---|---|---|---|---|
| **OUTCOME: PATIENT SURVIVAL** | | | | | |
| CIT group 2 (ref: CIT group 1) | -0.537 | 0.584 | 0.20 – 1.58 | 0.300 | 1.78 |
| **CIT group 3 (ref: CIT group 1)** | 1.123 | **3.075** | 1.40 – 7.15 | **0.006** | 1.74 |
| Delayed graft function (DGF) | -0.063 | 0.939 | 0.41 – 1.99 | 0.874 | 1.34 |
| ASA category 3 (ref: ASA 1–2) | 0.927 | 2.528 | 0.89 – 10.62 | 0.129 | 2.96 |
| ASA category 4 (ref: ASA 1–2) | -0.210 | 0.810 | 0.04 – 6.39 | 0.856 | 1.19 |
| Age >70 years (R) | -0.166 | 0.847 | 0.38 – 1.75 | 0.667 | 1.50 |
| Sex, female (R) | 0.025 | 1.026 | 0.47 – 2.50 | 0.952 | 2.80 |
| Postoperative infection | 0.109 | 1.116 | 0.56 – 2.25 | 0.757 | 1.87 |

| C Predictor Variable | $\beta$ | HR | 95% CI | p-value | VIF |
|---|---|---|---|---|---|
| **OUTCOME: PATIENT SURVIVAL** | | | | | |
| Delayed graft function (DGF) | -0.055 | 0.947 | 0.41 – 1.99 | 0.890 | 1.32 |
| DR mismatch 1 (ref: MM DR 0) | -0.508 | 0.602 | 0.23 – 1.69 | 0.313 | 2.35 |
| DR mismatch 2 (ref: MM DR 0) | -0.290 | 0.749 | 0.32 – 1.96 | 0.526 | 2.75 |

| C Predictor Variable | β | HR | 95% CI | p-value | VIF |
|---|---|---|---|---|---|
| CIT group 2 (ref: CIT group 1) | -0.525 | 0.592 | 0.20 – 1.60 | 0.310 | 1.81 |
| **CIT group 3 (ref: CIT group 1)** | 1.062 | **2.893** | 1.33 – 6.65 | **0.009** | 1.55 |
| ASA category 3 (ref: ASA 1–2) | 0.922 | 2.515 | 0.89 – 10.52 | 0.129 | 3.77 |
| ASA category 4 (ref: ASA 1–2) | -0.273 | 0.761 | 0.04 – 5.98 | 0.813 | 1.15 |

| D Predictor Variable | β | HR | 95% CI | p-value | VIF |
|---|---|---|---|---|---|
| **OUTCOME: PATIENT SURVIVAL** | | | | | |
| Delayed graft function (DGF) | -0.053 | 0.948 | 0.41 – 1.99 | 0.893 | 1.32 |
| Total mismatches (MM) | -0.048 | 0.953 | 0.74 – 1.25 | 0.719 | **5.31** |
| CIT group 2 (ref: CIT group 1) | -0.530 | 0.588 | 0.20 – 1.59 | 0.306 | 1.77 |
| **CIT group 3 (ref: CIT group 1)** | 1.121 | **3.067** | 1.43 – 6.97 | **0.005** | 1.60 |
| ASA category 3 (ref: ASA 1–2) | 0.938 | 2.556 | 0.90 – 10.74 | 0.125 | 4.54 |
| ASA category 4 (ref: ASA 1–2) | -0.241 | 0.786 | 0.04 – 6.15 | 0.835 | 1.25 |

**Table S6. Sensitivity analysis according to transplantation era.** Era 1: 1999-2006, Era 2: 2007-2013, Era 3: 2014-2019. Multivariate Cox regression analysis including era and Likelihood ratio test (Era x CIT interaction). ASA = American Society of Anesthesiologists; CIT = Cold Ischemia Time; R = Recipient.

| Predictor Variable | Hazard Ratio | 95% CI | p-value |
|---|---|---|---|
| **OUTCOME: PATIENT SURVIVAL** | | | |
| Era 2: 2007-2013 (ref: Era 1: 1999-2006) | 1.69 | 0.55 – 5.14 | 0.351 |
| Era 3: 2014-2019 (ref: Era 1: 1999-2006) | 2.59 | 0.81 – 8.30 | 0.107 |
| Rank (rank 2 vs rank 1) | 0.86 | 0.26 – 3.05 | 0.803 |
| CIT group 2 (ref: CIT group 1) | 0.86 | 0.18 – 3.73 | 0.843 |
| **CIT group 3 (ref: CIT group 1)** | **4.81** | 1.21 – 19.99 | **0.025** |
| Delayed graft function (DGF) | 1.28 | 0.46 – 3.37 | 0.624 |
| ASA 3 (ref: ASA 1–2) | 4.41 | 0.85 – 80.88 | 0.083 |
| ASA 4 (ref: ASA 1–2) | 2.39 | 0.09 – 64.12 | 0.557 |
| Age at transplantation >70 years (R) | 0.82 | 0.28 – 2.14 | 0.698 |
| Female sex (R) | 1.27 | 0.42 – 4.70 | 0.691 |
| Postoperative infection | 1.45 | 0.58 – 3.76 | 0.433 |

| Era x CIT interaction Likelihood ratio test | p-value | Significant era effect? |
|---|---|---|
| Patient survival | p=0.583 | No |
| Graft survival | p=0.254 | No |

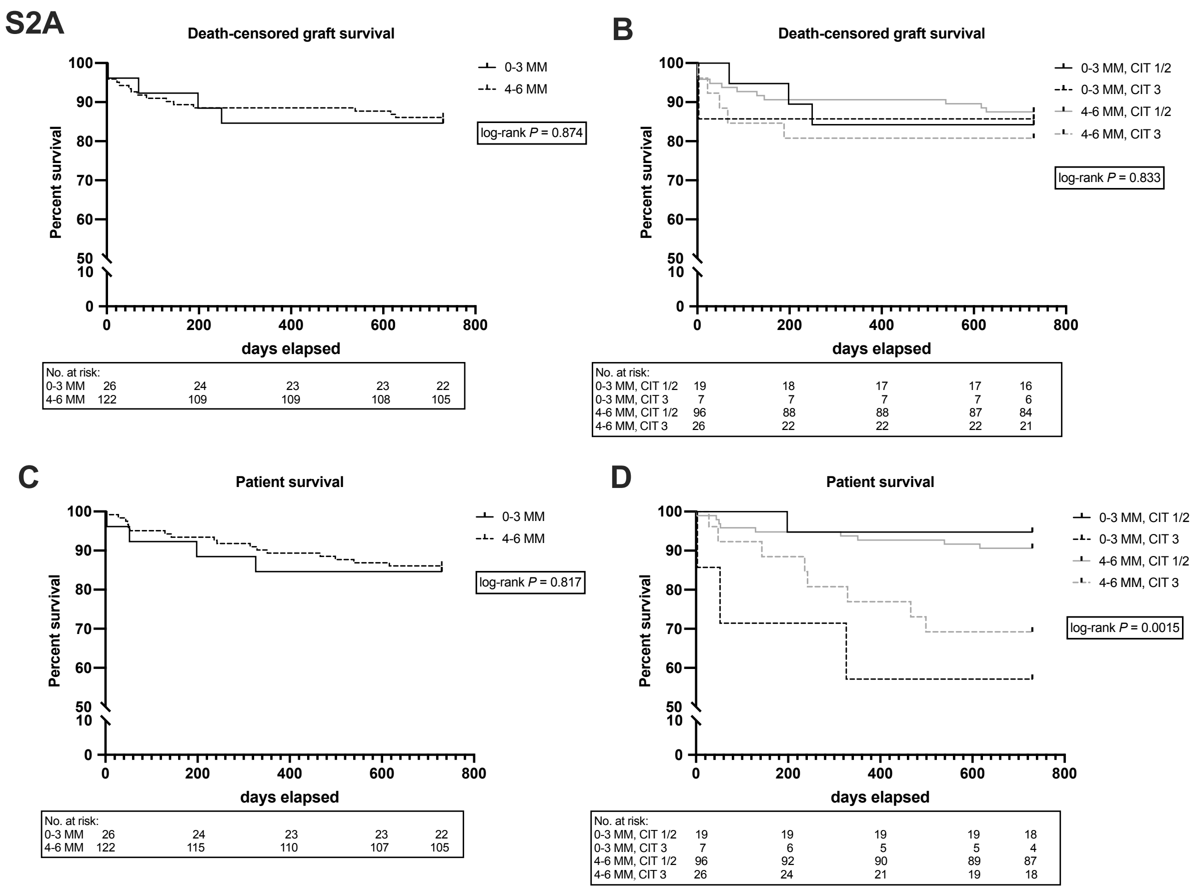


**Figure S4. Allograft and patient survival within the first two years post-KTx based on number of mismatches and CIT for 74 paired kidneys. (A)** death-censored graft survival by mismatches. **(B)** death-censored graft survival by mismatches and CIT groups. **(C)** patient survival by mismatches. **(D)** patient survival by mismatches and CIT groups. Kaplan-Meier graph with log-rank test used for graft loss and death. CIT1/2: cold ischemia time ≤ 12 h. CIT 3: cold ischemia time > 12 h. KTx: kidney transplantation. MM: mismatches.

| Outcome | Comparison | n (group A vs B) | Events / n | Effect Size | Power | Adequacy |
|---|---|---|---|---|---|---|
| Delayed Graft Function | CIT 3 vs CIT 1 | 54 vs 76 | 29 / 130 | h = 0.37 | **55.7%** | **Below 80%** |
| | CIT 3 vs CIT 2 | 54 vs 78 | 33 / 132 | h = 0.25 | **29.5%** | **Below 80%** |
| | CIT 2 vs CIT 1 | 78 vs 76 | 28 / 154 | h = 0.12 | **11.9%** | **Below 80%** |
| Patient Survival | CIT 3 vs CIT 1 | 54 vs 76 | 80 / 130 | HR = 1.74 | **68.3%** | **Below 80%** |
| | CIT 3 vs CIT 2 | 54 vs 78 | 76 / 132 | HR = 1.74 | **65.9%** | **Below 80%** |
| Death-Censored Graft Survival | CIT 3 vs CIT 1 | 54 vs 76 | 64 / 130 | HR = 1.38 | **24.6%** | **Below 80%** |
| | CIT 3 vs CIT 2 | 54 vs 78 | 58 / 132 | HR = 1.38 | **22.7%** | **Below 80%** |

**Table S7. Post-Hoc Power Analysis.** Post-hoc power analysis for the three primary outcomes. Total cohort: n = 208 patients. Power calculated using the Schoenfeld formula for survival outcomes and Cohen's h for DGF (proportion comparison). Alpha = 0.05 (two-sided) for all analyses. DGF = Delayed Graft Function. HR = Hazard Ratio (from Cox proportional hazards model; reference: CIT group 1). h = Cohen's h effect size for proportion comparisons. Events/n = number of events (DGF, deaths, or graft losses) / patients in the two compared groups. Adequate = power ≥ 80% (conventional threshold). Bonferroni-corrected significance threshold for three pairwise comparisons: $p < 0.017$.

# Table S8. STROBE Statement — Checklist of Items to be Included in Reports of Cohort Studies

| Item No | Recommendation & Reporting in This Manuscript | Page / Location |
|---|---|---|
| **Title and abstract** | | |
| **1** | **(a) Indicate the study's design with a commonly used term in the title or the abstract**<br>→ The title states the study design implicitly through the clinical context. The abstract (p.2) describes this as a retrospective cohort analysis using a paired-kidney design within the ESP framework.<br>**(b) Provide in the abstract an informative and balanced summary of what was done and what was found**<br>→ Abstract (200 words, p.2): reports design (retrospective, n=208), paired cohort (n=74 pairs), primary finding (CIT >12h: adjusted OR 6.3 for DGF, adjusted HR 3.19 for mortality), and clinical conclusion. | *Title page, p.1*<br>*Abstract, p.2* |
| **Introduction — Background / rationale** | | |
| **2** | Explain the scientific background and rationale for the investigation being reported<br>→ Introduction details: (i) ageing dialysis population and 5x mortality on waiting list vs age <50; (ii) ESP reduces waiting time >1.5 years and doubles life expectancy vs dialysis; (iii) CIT as crucial determinant of graft outcome, especially in aged kidneys; (iv) ischemia-reperfusion injury mechanism; (v) clinical observation at our center that consecutive transplantation may not disadvantage the second recipient — motivating a systematic investigation. | *Introduction*<br>*p. 3* |
| **Introduction — Objectives** | | |
| **3** | State specific objectives, including any prespecified hypotheses<br>→ Stated objective: to investigate the impact of CIT on DGF and long-term patient and graft outcomes using data from ESP patients receiving kidneys from the same donor, thereby eliminating individual kidney-level confounders. Prespecified hypothesis: CIT, rather than transplantation rank, is the primary determinant of outcome. | *Introduction*<br>*p. 3* |
| **Methods — Study design** | | |
| **4** | Present key elements of study design early in the paper<br>→ Described as monocentric retrospective cohort study with a paired-kidney case-control design. Both kidneys from 74 donors consecutively transplanted at one center; pairs differ primarily in CIT. An additional 60 single-kidney recipients included in secondary analyses (n=208 total). | *Methods*<br>*p. 4* |
| **Methods — Setting** | | |
| **5** | Describe the setting, locations, and relevant dates, including periods of recruitment, exposure, follow-up, and data collection<br>→ Single center: Freiburg Transplant Center, University Medical Center Freiburg, Germany.<br>→ Recruitment: 27 June 1999 – 27 August 2019 (20 years). | *Methods*<br>*p. 4* |

| Item No | Recommendation & Reporting in This Manuscript | Page / Location |
|---|---|---|
| | → All patients received ESP-allocated kidneys from deceased DBD donors ≥65 years.<br>→ Mean follow-up: 5.7 years<br>→ Data collection: retrospective from nephrological transplant outpatient clinic and local nephrologists. | |
| **Methods — Participants** | | |
| 6 | **(a) Give the eligibility criteria, and the sources and methods of selection of participants. Describe methods of follow-up**<br>→ Inclusion: all patients aged ≥65 receiving a deceased-donor kidney via ESP at Freiburg Transplant Center, 1999–2019. All donors aged ≥65 (DBD only; DCD not eligible under ESP during study period).<br>→ Follow-up: in-house nephrological transplant outpatient clinic and local nephrologists; all outcomes had complete data with no loss to follow-up<br>**(b) For matched studies, give matching criteria and number of exposed and unexposed**<br>→ Matching: inherent by donor identity (74 pairs). Rank 1 (shorter CIT, n=74) vs Rank 2 (longer CIT, n=74). Mixed logistic regression with random donor intercept accounts for within-pair clustering (ICC=0.22). | *Methods pp. 4-5* |
| **Methods — Variables** | | |
| 7 | Clearly define all outcomes, exposures, predictors, potential confounders, and effect modifiers. Give diagnostic criteria, if applicable<br>→ Primary outcome: DGF — need for ≥1 dialysis within 7 days post-transplant.<br>→ Secondary outcomes: death-censored graft survival; patient survival; graft loss (permanent return to dialysis).<br>→ PNF: permanent graft dysfunction without recovery; excluded from DGF analyses.<br>→ Exposure: CIT categorized as CIT 1 (0–8h), CIT 2 (8–12h), CIT 3 (≥12h).<br>→ Confounders: donor age, BMI, diabetes mellitus, hypertension, coronary heart disease (donor-level).<br>→ Other variables: rank, HLA mismatches (total, A, B, DR), ASA category, recipient comorbidities, warm ischemia time. | *Methods pp. 4-6* |
| **Methods — Data sources / measurement** | | |
| 8* | For each variable of interest, give sources of data and details of methods of assessment. Describe comparability of assessment methods if there is more than one group<br>→ CIT: prospectively recorded at transplantation (organ cold perfusion to vascular reperfusion).<br>→ DGF/PNF: in-hospital medical records (dialysis logs).<br>→ Graft loss/death: transplant outpatient records and mandatory Eurotransplant reporting.<br>→ Donor data: Eurotransplant allocation documentation.<br>→ HLA typing: standard serology/molecular methods per Eurotransplant protocol. | *Methods pp. 4-6* |

| Item No | Recommendation & Reporting in This Manuscript | Page / Location |
|---|---|---|
| | → ABO/HLA compatibility: all ABO-compatible; negative crossmatch mandatory throughout.<br>→ Missing data: <4% for any variable (primary immunosuppression (n=2, 0.96%), creatinine at discharge (n=7, 3.37%), warm ischemia time (n=1, 0.48%), time on dialysis before KTx (n=1, 0.48%), time on wait list (n=1, 0.48%), due to incomplete historical records). | |
| **Methods — Bias** | | |
| **9** | Describe any efforts to address potential sources of bias<br>→ Paired-kidney design inherently controls donor-level characteristics, isolating CIT as primary exposure.<br>→ Mixed logistic regression (random donor intercept) accounts for within-pair correlation.<br>→ Adjustment for donor comorbidities (diabetes, hypertension, CHD, age, BMI) minimizes confounding.<br>→ New Table 1B confirms no significant differences in recipient characteristics across CIT groups supporting absence of selection bias.<br>→ Era-based sensitivity analysis (1999–2006, 2007–2013, 2014–2019) addresses temporal bias; no significant Era × CIT interaction. | *Methods pp.4-6*<br>*Results pp.8–10* |
| **Methods — Study size** | | |
| **10** | Explain how the study size was arrived at<br>→ Retrospective inclusion of all eligible ESP recipients at the center over 20 years (n=208); no a priori sample size calculation.<br>→ Post-hoc power analyses: DGF (primary): 56% power; Mortality: 68% power; Graft loss: 25% power (underpowered, acknowledged as limitation). | *Methods p.4*<br>*Supp. Table S7* |
| **Methods — Quantitative variables** | | |
| **11** | Explain how quantitative variables were handled in the analyses. If applicable, describe which groupings were chosen and why<br>→ CIT categorized into 3 groups (0–8h, 8–12h, ≥12h) based on cohort median (9.35h) and clinical practicability. ROC analysis confirmed 12h optimal (Youden index (DGF): 8.5 h; AUC 0.592, p=0.031).<br>→ Normality tested by Shapiro-Wilk test for all continuous variables; non-normal distributions (CIT, BMI, dialysis time, creatinine) summarized as median (95% CI) and analyzed with non-parametric tests. | *Methods p.6*<br>*Supp. Table S1*<br>*Supp. Fig. S1 and S2* |
| **Methods — Statistical methods** | | |
| **12** | **(a) Describe all statistical methods, including those used to control for confounding**<br>→ Mixed effects logistic regression (GLMM, random donor intercept, lme4 R package) for DGF in 74 pairs. Cox proportional hazards for survival. Kaplan-Meier with log-rank test. Mann-Whitney U, Kruskal-Wallis, Fisher exact as appropriate. All two-sided; α=0.05. Analyses: R 4 and GraphPad Prism 11.<br>**(b) Describe any methods used to examine subgroups and interactions** | *Methods pp. 4–7* |

| Item No | Recommendation & Reporting in This Manuscript | Page / Location |
|---|---|---|
| | → Subgroups: DGF by CIT × rank; survival stratified by DGF status; HLA mismatch × CIT. Era-based sensitivity analysis with formal Era × CIT interaction test (LRT).<br>**(c) Explain how missing data were addressed**<br>→ Complete case analysis (<4% missing). PNF (n=15) excluded from DGF analyses to prevent misclassification.<br>**(d) Loss to follow-up**<br>→ No loss to follow-up for primary endpoints (mandatory Eurotransplant reporting).<br>**(e) Sensitivity analyses**<br>→ Era-based analysis; continuous CIT; PNF inclusion/exclusion; ROC-derived cut-point. | |
| **Results — Participants** | | |
| **13*** | **(a) Report numbers of individuals at each stage of study**<br>→ 134 donors; 74 pairs (148 recipients: Rank 1 n=74, Rank 2 n=74); 60 single-kidney recipients; total 208 patients. CIT groups: CIT 1 n=76, CIT 2 n=78, CIT 3 n=54. Flow diagram: Figure 1.<br>**(b) Give reasons for non-participation at each stage**<br>→ All consecutive ESP recipients included; no exclusions beyond ESP allocation criteria.<br>**(c) Consider use of a flow diagram**<br>→ Figure 1 presents complete study profile. | *Results p.8*<br>*Figure 1* |
| **Results — Descriptive data** | | |
| **14*** | **(a) Give characteristics of study participants and information on exposures and potential confounders**<br>→ Table 1A: Rank 1 vs Rank 2 (n=74 each). Table 1B: baseline by CIT group. Table 3A: donor characteristics. Only CIT differs significantly between ranks (p<0.0001); no significant differences in demographics, comorbidities, or HLA mismatches across CIT groups.<br>**(b) Indicate number of participants with missing data for each variable of interest**<br>→ primary immunosuppression (n=2, 0.96%), creatinine at discharge (n=7, 3.37%), warm ischemia time (n=1, 0.48%), time on dialysis before KTx (n=1, 0.48%), time on wait list (n=1, 0.48%) (due to incomplete historical records). All outcome variables: complete.<br>**(c) Summarize follow-up time**<br>→ Mean follow-up: 5.7 years | *Results pp.8*<br>*Tables 1, 3A* |
| **Results — Outcome data** | | |
| **15*** | Report numbers of outcome events or summary measures over time<br>→ DGF: 45/208 (21.6%); excluding PNF: 45/193 (23.3%). By CIT: 16.7% / 21.6% / 36.2%.<br>→ PNF: 15/208 (7.2%), similar across groups.<br>→ Graft loss: earlier in CIT 3 (median 57 vs 674 days, p=0.042).<br>→ Deaths: 5-year survival: CIT 1 69.5%, CIT 2 69.4%, CIT 3 56.7% (p<0.0001). | *Results pp.8-10*<br>*Tables 2, 4, 6*<br>*Figures 2, 3, 4* |

| Item No | Recommendation & Reporting in This Manuscript | Page / Location |
|---|---|---|
| | → Death-censored graft survival at 5 years: CIT 1 ~78%, CIT 2 ~83%, CIT 3 ~67% (p=0.345, NS). | |
| **Results — Main results** | | |
| **16** | **(a) Give unadjusted estimates and, if applicable, confounder-adjusted estimates and their precision**<br>→ DGF: adjusted OR CIT 3 vs CIT 1: 6.3 (95% CI 1.52–26.06, p=0.011). Donor age: OR 1.23/year (p=0.01). Adjusted for donor DM, CHD, hypertension, age, BMI.<br>→ Graft survival: DGF only independent predictor (adjusted HR 3.71, 95% CI 1.14–12.53, p=0.029).<br>→ Patient survival: CIT >12h adjusted HR 2.98 (95% CI 1.34–6.97, p=0.009).<br>**(b) Report category boundaries when continuous variables were categorized**<br>→ CIT 1: 0–8h; CIT 2: >8–12h; CIT 3: >12h. Confirmed by ROC (optimal cut-point 8.5h and 11.3h).<br>**(c) Consider translating estimates of relative risk into absolute risk**<br>→ 5-year survival: 69.5% (CIT1) vs 56.7% (CIT3) — absolute difference 12.8%. With DGF: 67.5% vs 23.5% — absolute difference 44%. | *Results pp.9-10*<br>*Tables 3B, 4, 6* |
| **Results — Other analyses** | | |
| **17** | Report other analyses done — e. g. subgroups and interactions, and sensitivity analyses<br>→ Survival stratified by DGF status (Figures 3B, 4B); by HLA mismatch × CIT (Figure 5); by rank (Figure S3, S4).<br>→ Sensitivity: rank as predictor (NS, Table S3); era-based analysis (No Era × CIT interaction, p=0.764, Table S6)<br>→ HLA mismatch: no survival difference by count alone; CIT >12h predicted worse survival regardless of mismatch group (log-rank p=0.0002, Figure 5D). | *Results pp.9-11*<br>*Figures 3–5*<br>*Supp. S3, S4, S6* |
| **Discussion — Key results** | | |
| **18** | Summarize key results with reference to study objectives<br>→ CIT is the primary determinant of DGF, graft failure, and patient survival in ESP kidney transplantation — over transplantation rank and HLA mismatch. CIT >12h: ~6-fold DGF risk (OR 6.3) and 3-fold mortality risk (HR 2.98). Rank 2 recipients not disadvantaged if CIT <12h. HLA mismatch count did not independently affect outcomes. | *Discussion p. 12* |
| **Discussion — Limitations** | | |
| **19** | Discuss limitations of the study, taking into account sources of potential bias or imprecision<br>1. Single-center retrospective design: limits generalizability.<br>2. European (mainly Caucasian) population.<br>3. 20-year period: era-related practice changes (mitigated by era sensitivity analysis, no significant interaction). | *Discussion pp. 15-16* |

| Item No | Recommendation & Reporting in This Manuscript | Page / Location |
|---|---|---|
| | 4. Sample size: underpowered due to inherently limited patient volume<br>5. DBD donors only — findings may not apply to DCD kidneys.<br>6. Static cold storage only — no HMP; results may differ with machine perfusion.<br>7. Generalizability limited to Eurotransplant/ESP systems. | |
| **Discussion — Interpretation** | | |
| **20** | Give a cautious overall interpretation of results considering objectives, limitations, multiplicity of analyses, results from similar studies, and other relevant evidence<br>→ Reducing CIT to <12h is the most actionable modifiable risk factor in ESP transplantation. Paired-kidney design provides robust evidence by eliminating donor confounding. Findings extend prior multicenter data (Frei et al. 2008: 3% graft loss/CIT hour) by quantifying a specific mortality threshold. Dose-response across both categorical and continuous analyses, biological plausibility, and consistency across eras support causal interpretation. Results must be interpreted cautiously given single-center retrospective nature and power limitations for rare outcomes. | *Discussion pp. 16* |
| **Discussion — Generalizability** | | |
| **21** | Discuss the generalizability (external validity) of the study results<br>→ Directly applicable to Eurotransplant-affiliated centers using ESP with DBD donors. The 12h CIT threshold offers a concrete operational decision point. Generalizability limited to: DCD donors (different CIT sensitivity); non-Eurotransplant systems (UNOS, UK); younger populations outside ESP criteria. Nationwide HMP rollout in Germany (DSO, Jan 2026) may alter absolute thresholds — prospective re-evaluation warranted. | *Discussion pp. 15-16* |
| **Other information — Funding** | | |
| **22** | Give the source of funding and the role of the funders for the present study<br>→ No external funding received. Authors declare no conflicts of interest. Statistical consultation provided by IMBI Freiburg (Institute of Medical Biometry and Statistics, University of Freiburg). | *Acknowledgements p. 17* |

*** Recommendations apply separately for exposed and unexposed groups.** *DBD = donation after brain death; CIT = cold ischemia time; DGF = delayed graft function; PNF = primary non-function; ESP = Eurotransplant Senior Program; HMP = hypothermic machine perfusion; ICC = intraclass correlation coefficient; LRT = likelihood ratio test; NS = not significant.*
